# Designing effective explainable AI: a human-centered evaluation of explanation formats in financial decision-making

**DOI:** 10.3389/frai.2026.1668029

**Published:** 2026-03-05

**Authors:** Henry Maathuis, Marcel Stalenhoef, Sieuwert van Otterloo, Raymond Zwaal, Kees van Montfort, Danielle Sent

**Affiliations:** 1 Research Group Artificial Intelligence, HU University of Applied Sciences Utrecht, Utrecht, Netherlands; 2 Jheronimus Academy of Data Science, Tilburg University, Eindhoven University of Technology, ‘s-Hertogenbosch, Netherlands; 3 Research Group Human Experience & Media Design, HU University of Applied Sciences Utrecht, Utrecht, Netherlands; 4 Center for Financial Innovation, Amsterdam University of Applied Sciences, Amsterdam, Netherlands

**Keywords:** explainable AI, explanation formats, finance, graphical design, human-centered evaluation

## Abstract

As artificial intelligence (AI) systems are increasingly deployed in high-risk financial decision-making contexts, the demand for transparency and interpretability becomes critical. Explainable AI (XAI) has emerged as a key research domain addressing these needs. While most existing XAI studies emphasize objective quality measures such as correctness and completeness of explanations, they often overlook the role of end-user requirements and the broader ecosystem of stakeholders. This study presents a human-centered evaluation of different visual explanation designs in financial AI applications, assessing their effectiveness. A two-phase mixed-method evaluation was conducted, combining user studies with end-users and a stakeholder workshop, to rank visual prototypes across four explanation types: feature importance, counterfactuals, contrastive/similar examples, and rule-based explanations. A key finding is the divergence between end-users and other stakeholders—including compliance officers, XAI consultants, and developers—with end-users indicating a preference for concise, contextually visual explanations (e.g., small sets of decision rules or risk plots relative to similar cases), while other stakeholders often favor more complete, technically detailed representations. This highlights a critical trade-off between interpretability and completeness. This suggests that visual encoding choices may affect the effectiveness of AI explanations across different stakeholder groups.

## 1 Introduction

Artificial Intelligence (AI) has become an increasingly important component of modern decision-making systems, influencing a wide range of industries, including healthcare and finance. As AI-driven models and solutions grow more sophisticated, their underlying complexity has raised significant concerns around transparency, interpretability, and ultimately, the trustworthiness of AI-generated outcomes (Phillips-Wren, 2012; Hawley et al., 1990; Amann et al., 2020). In particular, black-box models—characterized by opaque and non-intuitive decision processes—pose serious challenges for regulators, businesses, and end-users seeking to understand, challenge, or justify algorithmic outcomes (Hassija et al., 2024).

These concerns have fueled the emergence of Explainable Artificial Intelligence (XAI), a growing research field dedicated to improving the interpretability of AI systems and ensuring that decisions are comprehensible, justifiable, and actionable (Miller, 2019; Arrieta et al., 2020). The importance of explainability is further reflected in ethical and regulatory frameworks such as the Ethics Guidelines for Trustworthy AI (High-Level Expert Group on Artificial Intelligence., 2019), which identify it as a key pillar for fostering transparency and accountability in AI systems.

Although XAI has made considerable technical progress, much of the research continues to emphasize explanation generation rather than its reception and practical utility in real-world contexts (Mohseni et al., 2021). The presence of an explanation alone does not ensure that users will understand or trust the underlying system. For explanations to be effective, they must align with the audience's cognitive abilities, expectations, and prior knowledge (Brennen, 2020; Bertrand et al., 2022; Liao et al., 2020). This requires not only accurate content but also careful attention to how explanations are presented. Modalities such as textual descriptions, visual illustrations, or auditory feedback each influence how users process information. Interactive elements, in particular, can serve as scaffolding mechanisms, helping users to explore, navigate, and interpret AI outputs in more meaningful ways (Liao et al., 2020).

Despite this, the design of explanations—especially in terms of visual layout, modality, and interactivity—remains an underexplored dimension in XAI research. While some studies have examined how presentation formats affect comprehension and trust, few have systematically investigated the role of design in shaping user engagement and experience. This is a critical omission, as design fundamentally influences whether explanations are not just available but actually usable. Research in human-computer interaction (HCI) suggests that well-designed explanations can improve trust, enhance decision-making accuracy, and increase user satisfaction (Ehsan et al., 2021; Kaur et al., 2020). Yet, in many XAI studies, design remains secondary to algorithmic transparency and technical performance (Gilpin et al., 2018).

The need for explainable AI is particularly acute in high-risk domains such as finance, where AI systems are increasingly used for credit scoring, risk evaluation, and fraud detection. In such applications, explainability is essential not only for regulatory compliance and auditability but also for ensuring fair, informed, and transparent decision-making. The proposed EU AI Act underscores this requirement by categorizing AI systems into risk tiers and imposing stringent transparency requirements on high-risk applications (European Commission, 2021). Furthermore, recent work emphasizes that effective explanations must be actionable, narratively coherent, and context-sensitive in order to support meaningful human-AI collaboration (Kim et al., 2024b). A large-scale study by the European Commission's Joint Research Centre further highlights the importance of human oversight, showing its role in mitigating discriminatory outcomes in AI-assisted decision-making (Gaudeul et al., 2025).

In the financial sector, the diversity of stakeholders—including regulators, compliance officers, analysts, and end-users—creates an additional challenge for explainability. Each group may have distinct informational needs, interpretive skills, and decision contexts. For instance, a financial analyst may require a detailed feature-level explanation, whereas a loan applicant may only need a clear, high-level rationale for the outcome (Langer et al., 2021). Current XAI methods often fall short of addressing this spectrum of needs, pointing to a critical gap in adaptive, stakeholder-specific explanation strategies (Kim et al., 2024c).

Moreover, the evaluation of XAI techniques is frequently limited to algorithmic metrics or controlled lab benchmarks rather than real-world user assessments (Doshi-Velez and Kim, 2017; Lipton, 2018). Studies on end-user perceptions reveal a recurring mismatch between what researchers define as “explainable” and what users actually find understandable or useful (Liao et al., 2022). A recent systematic review by Belle and Papantonis (2021) echoes these concerns, calling for more empirical user-centered research. Additionally, psychological and cognitive factors that shape how users engage with explanations remain insufficiently addressed in current evaluation frameworks (Ali et al., 2023).

To address these limitations, our study conducts a human-centered evaluation of visual presentation formats for communicating explainable AI (XAI) outputs in the financial domain, framed as an exploratory analysis due to the small sample size. We adopt a model-agnostic approach, focusing not on generating explanations from specific algorithms but on how different types of pre-defined explanations—such as feature importance or counterfactuals—can be effectively presented and understood. Our investigation centers on two real-world use cases: (1) business credit provision and (2) automotive insurance claim fraud assessment. Since all participating end-users already work directly with these AI-supported decision processes, they possess an inherent understanding of the underlying use case, decision task, and role of the AI system. The visualizations and explanation designs were therefore aligned with their existing domain knowledge and everyday workflows. End-users evaluated explanations on explicitly given dimensions such as usefulness, clarity, and actionability, which reflect meaningful engagement with the context rather than mere visual appeal, and generally rated the designs positively, suggesting they supported understanding and decision-making within their specific workflows (Kim et al., 2024b).

Through this research, we aim to generate actionable insights for AI developers, financial institutions, and policymakers working toward more transparent, trustworthy, and user-centered AI systems. By bridging the persistent gap between technical explainability and real-world usability—particularly through a focus on visual and interaction design— we seek to advance the development of AI systems that are not only interpretable, but also intelligible and impactful in practice.

## 2 Materials and methods

In this study, we design and evaluate the effectiveness of various designs of explanations for different explanation types. To achieve this, we first distill a set of requirements that a meaningful explanation must meet based on existing literature, studying two real-world use-cases and studying rules and legislation; specifically, the considerations mentioned in the AI Act and GDPR (European Commission, 2024; European Union, 2016). We further elicit user needs through interviews with end-users from both use cases. Based on these requirements and insights, we iteratively developed and refined the explanation designs in close alignment with end-users and use-case owners. The final designs are provided in the Supplementary material.

Afterwards, we employ a two-phase evaluation process to assess the effectiveness of different visualization approaches for various explanation types. The first phase consists of user studies with end-users from our use-case partners, where we collect insights on user preferences and evaluate them on human-centered criteria (Kim et al., 2024a) through questionnaires. This feedback is then used to make small refinements to the explanation designs.

In the second phase, the evaluation extends to a wider range of stakeholders, including consultancy partners, compliance officers, and other professionals involved in XAI systems. This phase is conducted through a workshop, where participants rank the prototypes using the 100-dollar method for each explanation format, followed by qualitative discussions on the highest-ranked designs. By incorporating perspectives beyond end-users, this approach aims to keep the explanations both user-centric and aligned with broader stakeholder needs.

Therefore, the effectiveness of these designs is evaluated through mixed-method use-case studies with various stakeholders.

### 2.1 Law-based considerations

When designing high-risk AI systems, it is essential to consider legal requirements related to transparency and explainability. In the European context, key regulations such as the Artificial Intelligence Regulation (AI Act) (European Commission, 2024) and the General Data Protection Regulation (GDPR) (European Union, 2016) apply. Organizations must comply with both frameworks when deploying AI systems that affect individuals or process personal data.

The AI Act, which formally came into effect on August 1, 2024, establishes explicit obligations for high-risk AI systems. Section 86 and Recital 171 of the AI Act, specify that individuals subject to significant decisions based primarily on AI outputs are entitled to a clear and meaningful explanation, sufficient to understand the basis of the decision and exercise their rights. For instance, this applies to financial customers whose transactions are reviewed by an AI system. Recital 73 further requires that AI systems be designed to enable human operators, such as employees of a financial institution, to oversee the system's functioning, ensure correct usage, and mitigate adverse impacts. Providers are obliged to implement operational constraints and assign oversight responsibilities to competent personnel with the necessary training and authority, and to ensure that users possess sufficient literacy to understand explanations necessary for monitoring and intervention.

The GDPR complements the AI Act by regulating automated decision-making that involves personal data. Article 14 requires controllers to provide data subjects with meaningful information about the logic of automated decisions, their significance, and consequences, particularly when personal data were not obtained directly from the data subject. In financial contexts, this typically applies to bank customers, whereas the user of the system is the bank employee who first receives the explanation and may subsequently communicate it to the customer.

Legal requirements distinguish between global and local explanations. Global explanations provide insight into the AI system as a whole, including its architecture, training data, modeling techniques, testing procedures, and instructions for use, and can be embedded in help screens, manuals, or other accessible documentation. Local explanations, in contrast, pertain to individual decisions and describe specific inputs, intermediate factors or sub-scores, and the rationale behind a particular output. For example, a local explanation might indicate why a particular insurance claim was denied, referencing relevant features such as payment history or other criteria used by the AI system.

In this study, we focus on the requirements for local explanations. While global explanations remain important for user orientation, the design challenges and regulatory obligations are most pronounced in local explanations, which directly support decision-making and compliance. These legal considerations, together with insights from existing XAI literature, form the foundation for a comprehensive set of explanation requirements that can guide the design of practical, user-centered AI explanations as discussed in Section 2.2.

### 2.2 Consolidated set of explanation requirements

A comprehensive set of requirements was established by reviewing the literature on Explainable AI (XAI) (Kim et al., 2024a) and analyzing relevant legal frameworks, such as the GDPR and EU AI Regulation as described above. This theoretical foundation was further refined through expert workshops involving a total of seven academic and industry professionals, including XAI consultants, data scientists/developers of predictive financial models, and representatives from other financial bodies. This diverse group ensured that the requirements were both conceptually robust and practically relevant for financial applications. In the workshops, each requirement was assigned a score based on its priority, using a star-rating system in which a requirement could receive *zero, one*, or *two* stars. Each expert assigned their own rating, and the final score for a requirement was calculated as the average of all expert ratings. This averaging process produced fractional scores (e.g., 1.8), which represent the mean importance across experts. The star ratings were used to determine the prioritization of the requirements:

- A requirement with more stars than another indicates a higher priority.
- There are no restrictions on the number of requirements receiving two stars, meaning multiple requirements can be rated with the highest priority.

This ranked set of requirements was compiled into a list after the workshop and sorted by their importance. This final validated set of requirements is used as input for designing explanations for AI systems and is listed in Table 1.

**Table 1:** Final ranked requirements for local explanations.

**Rank**	**Requirement**	**Score**
1	Helps people understand how to change a decision (e.g., from reject to accept).	1.8
2	Encourages users to take action (e.g., look up or verify information).	1.7
3	Helps users recognize risks of bias, profiling, and discrimination.	1.7
4	Serves as a means for users to provide feedback to the model.	1.7
5	Enables users to explain the decision to the affected person.	1.5
6	Reduces over-reliance on AI outputs (prevents automation bias).	1.5
7	Supports oversight by helping users determine when not to use a high-risk AI system.	1.5
8	Must be compact and concise.	1.3
9	Increases user confidence in decision-making.	1.3
10	Helps detect anomalies, dysfunctions, and unexpected performance.	1.3
11	Provides interactivity, allowing users to engage with the explanation.	1.3

These requirements were used to inform the development of the visual prototypes, guiding the design of different visual explanation formats tailored to specific explanation types. Section 2.3 describes the user needs related to the investigated use-cases. In Section 2.4, we list general design considerations that were taken into account when designing the explanation formats.

### 2.3 User needs

To ensure that the explanation requirements were grounded in actual practice, we implemented a two-stage process to capture detailed use-case contexts and user needs.

In the first stage, use-case partners completed a structured use-case form that documented key aspects of their decision-support systems. This form gathered information on: (1) a comprehensive description of the use-case, (2) the types of decision-support systems in use (e.g., AI or rule-based systems), including specific techniques (e.g. logistic regression, XGBoost, rule-based methods, neural networks), (3) the operational history, function, and data flow (inputs/outputs) of these systems, (4) the processes supported by the systems and the steps involved, and (5) the stakeholders and their respective roles (e.g., customers, regulators, auditors). The specific use-case form can be found in the Supplementary material.

In the second stage, we conducted semi-structured interviews with end-users of these systems. The interview protocol began with the administration of an informed consent procedure and the collection of demographic data (including main role, tenure, and which role, if any, they had in the implementation of the system). Participants were then asked to describe in detail the decision-making process they follow when using the system, with prompts to explain the specific steps, challenges encountered, and the support provided by the system's outputs. Particular emphasis was placed on the nature of any explanations received: how outcomes are presented, the content and technical underpinnings of the explanations, and how the explanation is (visually) communicated. The respondents were also asked to indicate their needs and suggestions for improving the explanations as part of their decision support systems. The interview protocol is attached in the Supplementary material.

The data obtained from these interviews were analyzed and organized into user stories that were then integrated into the iterative development of visual prototypes for AI explanations. This approach ensured that the prototypes were aligned with both the technical requirements and the real-world challenges faced by users in the financial sector.

### 2.4 Prototype development

Drawing on insights from the literature, completed use-case forms, and user interviews, different visual prototypes were developed to illustrate the AI decision-making process. To ensure that the designs were meaningful, functional requirements were first derived from the interviews using user stories structured as:

*As a [user role], I want to [goal], so that [desired outcome]*.

In response to these user stories, concrete functional requirements were specified to guide the design and implementation of the explanation interfaces. Table 2 presents the main user stories identified across both use-cases, their implications for interface design, and the resulting functional requirements. These requirements served as a design blueprint for all subsequent prototypes and ensured that each explanation type addressed a clearly articulated user need.

**Table 2:** User stories and derived functional requirements.

**User story**	**Design impact**	**Functional requirements**
As a user, I want to see which features contributed most to the decision, so that I can understand the reasoning behind the outcome.	Feature importance visualization (e.g., bar chart)	Display the top five influential features; apply color coding for positive and negative contributions.
As a user, I want to view similar past cases and their outcomes, so that I can compare and validate the current decision.	Similar/contrastive cases examples	Show three to six similar cases; include both matching and contrasting outcomes.
As a user, I want to explore hypothetical changes to inputs, so that I can see what adjustments would lead to a different outcome.	Counterfactual explanations	Present original and modified outcomes side by side; suggest alternative values that would lead to acceptance.
As a user, I want to see which decision rules were applied and their impact, so that I can understand the logic behind the decision.	Rule-based explanations	List applied rules with their outcomes; highlight thresholds and actual values; allow users to expand rules for additional details.
As a user, I want a clear visual summary of the decision and explanation, so that I can easily communicate it to stakeholders.	Summary panel	Display decision score and risk category; apply concise explanatory text; use color cues to support fast interpretation.
As a user, I want the interface to clearly differentiate between neutral, uncertain, and high-risk outcomes, so that I can interpret the decision correctly.	Three-tier risk categorization	Provide consistent visual representations for neutral, uncertain, and high-risk outcomes; include explicit labels or legends; avoid implicit positive/negative outcome framing.
As a user, I want neutral indicators for non-risk values, so that I don't assume the outcome is automatically positive.	Semantically neutral visual encoding	Use neutral visual styles for non-risk values; clearly distinguish neutral, warning, and high-risk states; avoid color cues, such as green, implying approval.

For each use-case, designs were created for four explanation types: counterfactual explanations, feature importance explanations, similar/contrastive examples, and rule-based explanations. While most explanation types were represented by five designs each, the similar/contrastive examples category included only four designs for the business loan use-case and five for the insurance fraud use-case. The designs were largely consistent across use-cases but adapted to reflect the respective domain context. This resulted in a total of 20 prototypes for the business loan use-case and 19 for the car insurance fraud use-case, with the discrepancy due to a small mistake in the experimental setup. This is reflected with the dashes in the Similar/Contrastive Cases portion of Table 3.

**Table 3:** Ranking of prototypes across different explanation methods.

**Feature importance**						**Counterfactuals**					
	A	B	C	D	E		A	B	C	D	E
Participant 1.1	5	4	3	1	2	Participant 1.1	4	5	3	1	2
Participant 1.2	3	1	4	2	5	Participant 1.2	1	5	4	3	2
Participant 1.3	2	3	4	1	5	Participant 1.3	2	5	1	3	4
Total score	10	8	11	4	12	Total score	7	15	8	7	8
Participant 2.1	2	4	1	3	5	Participant 2.1	5	3	4	1	2
Participant 2.2	1	5	3	2	4	Participant 2.2	3	4	5	2	1
Participant 2.3	2	5	3	1	4	Participant 2.3	3	5	4	1	2
Total score	5	14	7	6	13	Total score	11	12	13	4	5
**(a)**						**(b)**					
**Rule-based explanations**						**Similar/contrastive cases**					
	A	B	C	D	E		A	B	C	D	E
Participant 1.1	5	1	4	3	2	Participant 1.1	4	3	1	2	–
Participant 1.2	2	1	5	4	3	Participant 1.2	3	1	2	4	–
Participant 1.3	5	4	3	2	1	Participant 1.3	3	4	2	1	–
Total score	12	6	12	9	6	Total score	10	8	5	7	–
Participant 2.1	2	1	5	4	3	Participant 2.1	4	1	3	5	2
Participant 2.2	3	1	5	4	2	Participant 2.2	4	5	2	3	1
Participant 2.3	1	2	5	4	3	Participant 2.3	5	4	3	2	1
Total score	6	4	15	12	8	Total score	13	10	8	10	4
**(c)**						**(d)**					

Each quadrant shows Participant scores and total scores for an explanation method. Gray highlights indicate lowest total scores representing the best ranked prototypes.

The design decisions were guided by well-established principles from cognitive science and human-computer interaction (HCI). From Cognitive Load Theory (Sweller, 1988), we applied two concrete principles. First, reducing extraneous cognitive load by removing non-essential visual elements and avoiding dense text blocks. Second, structuring intrinsic load by chunking related information into grouped sections within each explanation. These decisions aimed to support users in processing model outputs without being overloaded. Recent work highlighting the effects of presentation order and morphological clarity on cognitive load, trust, and confidence in AI systems further supports these choices (Hudon et al., 2021). Selective Attention Theory (Treisman and Gelade, 1980) guided our use of color, contrast, and spatial positioning to draw the viewer's attention to key explanatory elements—such as the primary feature contributions, decision rationale, or risk indicators—while de-emphasizing less important information.

**Figure 1:**
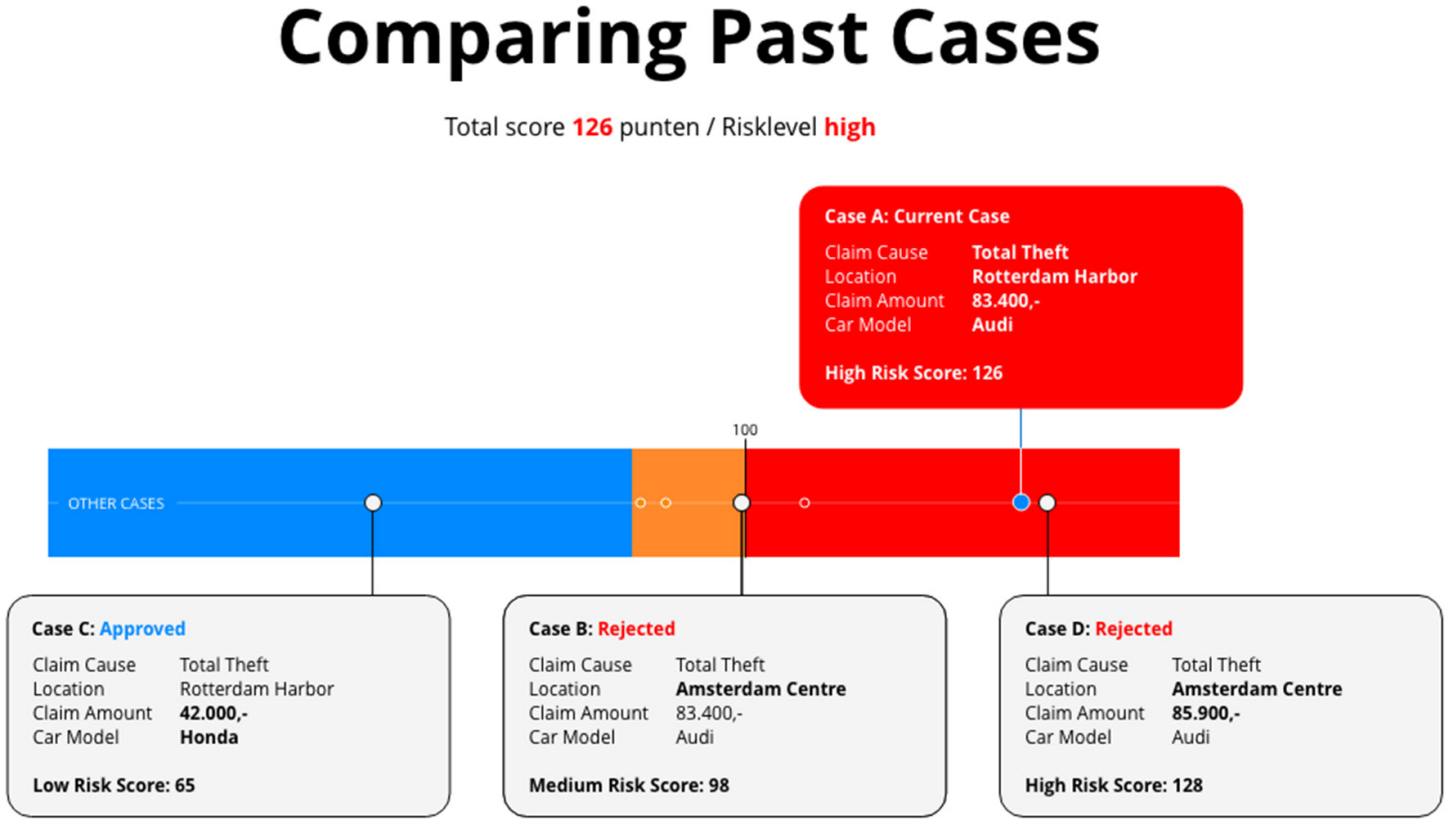
Contrastive/similar examples design A for the insurance fraud use-case.

Designing user interfaces depends not only on defined requirements but also on the implicit expertise, intuition, and aesthetic judgment of the graphic designer. These factors shape critical aspects such as layout, visual hierarchy, color schemes, and typographic choices, which are elements that can influence the interpretability and usability of interface explanations (Tidwell, 2010; Norman, 2013). To support transparency and reproducibility, all design prototypes developed in this study are included for reference. All 20 prototypes developed for the car-insurance fraud use case are available in the Supplementary material; Figure 1 illustrates a representative contrastive/similar example design.

### 2.5 Evaluating the visual designs with end-users

The evaluation of these prototypes with end-users was carried out through an exploratory quantitative approach. For each of the two use cases, three end-users (n = 6 total) completed all unique pairwise comparisons for each explanation type. Each explanation type included five different visualizations, resulting in 10 unique pairwise comparisons per explanation type per participant. From these selections, we derived a preference ranking from most to least preferred visualization per explanation type, with the design chosen most frequently across pairings representing the participant's top preference.

In the second stage, the end-users completed a 5-points Likert scale questionnaire assessing the prototypes based on 11 user-centered explanation criteria: *understanding, ease of understanding, ease of use, satisfaction, usefulness, typicality, sufficiency, subjective correctness, compactness*, and *actionability*. These criteria were distilled from prior research (Kim et al., 2024a). The complete questionnaire is attached in the Supplementary material.

The quantitative data, consisting of prototype selection rankings and questionnaire ratings, were analyzed to determine the most preferred prototype for each explanation type and to identify the key criteria influencing user preferences. Participants rated each prototype based on the 11 predefined criteria.

To assess the relationship between these criteria and prototype preference, we computed Spearman's rank correlation coefficient (ρ) between the binary preference variable (most preferred prototype per explanation type = 1, others = 0) and the corresponding questionnaire ratings. Spearman's ρ measures the strength and direction of a monotonic relationship between ordinal variables, making it appropriate for ranking-based data.

The exploratory analysis was conducted across two use-cases. In the credit loan use-case, participants evaluated 19 prototypes, resulting in a dataset of 3 × 11 × 19 = 627 observations, where each row represents a prototype evaluation based on the 11 criteria. In the insurance claim use-case, 20 prototypes were assessed, yielding 3 × 11 × 20 = 660 observations. Participants were not compensated for their participation.

Spearman's correlation was computed for each criterion to determine its predictive value in relation to prototype preference. Higher absolute values of ρ indicate a stronger relationship between a given criterion and user preference. The results are listed in Section 3.1.3.

### 2.6 Workshop to assess the generality of the visual designs

To complement the user studies, we conducted a workshop involving key stakeholders with expertise in explainable AI (XAI) systems. The workshop attracted four participants including two representatives of consultancy firms building explainable AI solutions for the financial sector, a compliance officer, and a developer/owner of XAI use-case systems. This broader group of stakeholders provided insights beyond end-user preferences, ensuring that the evaluation captured regulatory, practical, and implementation considerations relevant to XAI explanation design. Participants were not compensated for their participation.

The workshop followed a structured two-step mixed-method approach. In the first step, participants evaluated the same set of explanation designs as presented to the end-users, consisting of five designs for each of the four explanation types: feature importance, counterfactuals, similar/contrastive examples, and rule-based explanations. The prototypes used in Phase 2 were identical to those in Phase 1; no refinements were introduced between phases. This decision was intentional, as it ensured that differences in feedback or preferences could be attributed to stakeholder perspectives rather than changes in the materials, thereby enabling a more direct comparison across phases. The 100-dollar method was employed for this evaluation, requiring participants to allocate a virtual budget of 100 dollars across the designs within each explanation type. This forced-choice allocation method provides a quantitative measure of preference by revealing the relative value participants assign to different designs.

In the second step, the top three designs for each explanation type, as determined by the 100-dollar method, were subjected to a qualitative analysis. Participants provided feedback on the aspects of these top-ranked designs that contributed to their usability, clarity, and effectiveness. Additionally, they were asked to suggest modifications that could enhance the generality and adaptability of the designs across different XAI contexts and use-cases oriented on tabular data. This qualitative feedback aimed to ensure that the explanation visualizations were not only effective in specific scenarios but also applicable to a broader range of stakeholders and systems.

## 3 Results

### 3.1 User-result studies

This section presents the findings from the quantitative user studies, detailing the assessment of XAI prototypes by end-users on explanation design.

#### 3.1.1 Evaluation with end-users

To evaluate the XAI prototypes, two companies participated: a business loan provider and an insurance company. Each company had three participants involved in the evaluation. At the loan provider, underwriters assessed the prototypes, whereas at the insurance company, consultants evaluated them in the context of fraud detection. Participants completed a questionnaire, as outlined in Section 2.5, evaluating the prototypes based on 11 human-centered explanation criteria.

#### 3.1.2 Prototype ranking

For each of the four types of explanation, users were asked to indicate which of the different designs (A-E) was preferred. Table 3 shows the results of this evaluation.

From the **feature importance** quadrant (top-left) of Table 3, it can be seen that design D received the lowest total rank score from participant group 1 (score = 4), while design E was the least preferred (score = 12). In participant group 2, design A scored the best (score = 5), and design B had the lowest ranking (score = 14). In group 2, the difference in preference between designs A and D is only 1 point.

In terms of **counterfactuals**, as shown in the top-right quadrant of Table 3, designs A and D were the most preferred in participant group 1, while design B was the least preferred. In participant group 2, design D received the highest preference, while design C was the least favored. Overall, design D performed well across both groups, suggesting that it was perceived as a strong candidate for presenting counterfactual explanations.

Looking at **rule-based explanations** in the bottom-left quadrant of Table 3, participant group 1 most favored designs B and E, while designs A and D were the least preferred. In participant group 2, design B stood out as the favorite, while design C had the lowest preference score. The consistency in scores for design B suggests that it was well received for rule-based explanations.

For **similar/contrastive cases**, the bottom-right quadrant of Table 3 shows that participant group 1 preferred design C, despite design A receiving the highest overall score. In contrast, group 2 most favored design E, with design A being rated the lowest. As noted earlier, design E was not included in group 1's evaluation due to a small mistake in the experimental setup. When considering only the first four designs, both groups exhibited a shared preference for design C.

#### 3.1.3 Explanation criteria ratings

Table 4 provides an overview of how the different designs were rated based on the various criteria, derived from previous research (Kim et al., 2024a). Specifically, the properties related to the quality of the explanations were considered.

**Table 4:** For each criterion, the average score (1–5) for each prototype and the overall criterion average.

**FinTech Credit**	**Feature importance**					**Counter-factuals**					**Similar-contrastive**					**Rule-based explanations**					**Avg**.
	A	B	C	D	E	A	B	C	D	E	A	B	C	D	E	A	B	C	D	E	
Understand	3.7	4.0	3.7	4.3	3.0	4.3	4.0	3.3	4.3	3.0	2.7	2.7	4.0	3.0	—	3.7	4.3	3.7	4.0	4.3	3.7
Easy to understand	3.7	4.3	2.3	4.3	2.0	4.7	3.7	3.7	4.3	2.7	2.3	2.0	3.7	3.0	—	4.0	4.3	3.0	3.7	3.7	3.4
Easy to use	3.3	4.0	2.7	4.7	2.0	4.3	2.3	3.3	4.0	2.3	2.0	3.0	3.7	2.3	—	3.3	4.0	2.7	4.0	4.0	3.3
Satisfying	2.7	3.0	3.3	3.7	2.3	3.7	3.0	3.3	3.3	3.3	1.7	3.3	4.0	2.7	—	3.0	3.3	2.7	3.3	3.7	3.1
Useful	3.3	3.7	3.0	4.0	2.7	3.7	2.3	3.0	3.7	3.0	2.3	3.3	4.0	3.0	—	3.7	4.3	3.7	4.0	4.0	3.4
Trustworthy	3.7	3.7	3.7	4.3	3.7	4.0	3.3	4.0	3.7	4.0	3.3	3.7	4.3	3.7	—	3.7	4.7	4.3	4.0	4.0	3.9
Typical	3.7	3.7	2.7	3.7	2.3	3.7	3.0	3.7	3.3	2.7	3.0	3.7	3.7	3.0	—	3.7	4.3	4.0	4.0	3.7	3.4
Sufficient	2.3	3	2.7	3.3	2.0	3.0	2.3	4.0	4.0	2.7	1.7	3.3	2.3	3.0	—	2.7	4.3	3.7	4.0	4.0	3.1
Correct	4.0	4.3	3.7	4.0	3.0	4.3	3.7	4.0	4.0	4.0	3.0	3.7	4.3	3.7	—	3.3	4.3	3.7	4.0	4.3	3.9
Concise	3.7	4.3	3.7	4.0	2.3	4.3	2.7	2.3	4.7	3.0	3.3	2.7	3.7	2.0	—	4.0	4.0	2.0	2.7	3.3	3.3
Act	2.7	2.7	2.3	3.7	2.0	3.3	2.0	3.0	3.0	3.0	2.3	3.3	3.7	3.0	—	3.0	3.3	3.7	4.0	4.3	3.1
Avg.	3.3	3.7	3.1	4.0	2.5	3.9	2.9	3.4	3.8	3.1	2.5	3.2	3.7	2.9	—	3.5	4.1	3.4	3.8	3.9	
**Insurance claims**	**Feature importance**					**Counter-factuals**					**Similar-contrastive**					**Rule-based explanations**					**Avg**.
	A	B	C	D	E	A	B	C	D	E	A	B	C	D	E	A	B	C	D	E	
Understand	4.7	4.7	4.0	4.7	4.3	4.7	3.7	3.7	4.7	4.3	3.7	3.7	4.3	3.3	4.3	4.3	4.3	3.7	3.7	3.7	4.1
Easy to understand	4.7	4.3	4.0	4.3	3.0	4.0	3.0	3.0	4.3	4.0	2.3	3.0	4.0	2.7	4.7	3.7	4.3	3.0	3.0	3.7	3.7
Easy to use	4.7	3.7	3.7	4.3	3.0	4.0	3.3	3.3	4.3	4.0	3.0	3.0	4.3	3.0	4.7	3.7	4.3	3.3	3.3	3.7	3.7
Satisfying	4.0	3.0	3.7	4.3	2.7	3.7	3.0	3.7	5.0	4.3	3.3	3.7	4.3	3.3	4.7	3.3	4.3	3.7	3.0	3.7	3.7
Useful	4.3	3.7	3.0	3.7	3.0	3.0	3.3	3.3	4.7	4.0	3.7	3.3	4.0	3.7	3.7	3.7	4.3	3.7	3.7	3.3	3.7
Trustworthy	4.0	4.0	4.0	4.3	3.3	4.0	4.0	3.0	4.3	4.3	4.0	4.0	4.0	4.0	4.7	3.7	4.3	4.3	4.0	4.3	4.1
Typical	4.3	4.0	4.0	4.3	3.0	3.7	3.0	3.7	4.0	4.0	3.7	4.0	4.3	4.0	4.3	4.3	4.3	3.3	3.3	3.3	3.9
Sufficient	4.0	4.3	3.3	3.7	2.7	3.3	4.0	4.3	4.7	4.3	3.7	4.0	3.7	3.3	4.7	3.7	4.7	3.3	3.0	3.7	3.8
Correct	4.0	4.3	4.0	3.7	3.3	4.0	4.0	4.0	5.0	4.3	4.0	4.0	4.0	3.7	4.7	4.3	4.7	4.0	4.0	4.0	4.1
Concise	4.3	3.0	4.3	3.3	2.7	4.3	3.0	2.3	4.3	4.0	3.3	4.3	3.7	3.3	4.0	4.0	5	2.3	2.7	3.0	3.6
Act	3.3	3.0	3.0	3.3	3.0	3.3	3.3	3.3	3.7	3.3	2.7	3.3	3.3	3.0	3.3	3.3	3.3	3.0	3.0	3.0	3.2
Avg.	4.2	3.8	3.7	4	3.1	3.8	3.4	3.4	4.5	4.1	3.4	3.7	4.0	3.4	4.3	3.8	4.4	3.4	3.3	3.6	

The highest scores are marked in gray.

A Spearman rank correlation analysis was conducted to examine the relationship between explanation prototypes and user criteria for both the **Insurance Claims** and **FinTech Credit** use-case. Table 5 presents the correlation coefficients (ρ) for each criterion across the two use-cases. The results indicate that for **Insurance Claims**, the strongest correlation was observed for *Concise* (ρ = 0.56), followed closely by *Easy to Use* (ρ = 0.51). The correlation for *Satisfying* was also relatively high (ρ = 0.48), indicating that user satisfaction is closely linked to the preferred prototypes. For **FinTech Credit**, the highest correlation was found for *Satisfying* (ρ = 0.55), followed by *Easy to Use* (ρ = 0.50). Other notable correlations include *Sufficient* (ρ = 0.41) and *Correct* (ρ = 0.41).

**Table 5:** Spearman rank correlations for both use-cases.

**Criteria**	**ρ (Insurance claims)**	**ρ (FinTech credit)**
Understand	0.21	0.24
Easy to understand	0.44	0.34
Easy to use	**0.51**	**0.50**
Satisfying	0.48	**0.55**
Useful	0.30	0.20
Trustworthy	0.23	0.20
Typical	0.28	0.39
Sufficient	0.39	0.41
Correct	0.44	0.41
Concise	**0.56**	0.26
Act	0.34	0.40

Bold values indicate the strongest correlations observed in the table.

It is worth noting that in 10 of the 24 cases (41.7%) the design preferred by participants did not align with the design receiving the lowest aggregated criteria score (Table 6). While this discrepancy may partly reflect noise due to the small sample size, it also suggests that participants may weigh certain criteria differently when making overall preferences. In Company 2, the divergence was even more pronounced (8 of 12 cases, 75%), indicating that contextual factors could play a role in shaping XAI design preferences, though this interpretation should be treated as exploratory given the limited dataset.

**Table 6:** For each participant, the preferred design is presented (left) and the design based on the sum of scores for the individual characteristics (right).

	**Feature importance**		**Counter-factuals**		**Similar/ contrastive**		**Rule-based explanations**	
	**Pref**	∑	**Pref**	∑	**Pref**	∑	**Pref**	∑
Participant 1.1	D	D	D	D	C	C	B	B
Participant 1.2	B	D	A	A	B	B	E	E
Participant 1.3	D	D	A	D	D	D	E	E
Participant 2.1	C	A	D	E	B	E	B	B
Participant 2.2	A	C	E	D	E	B	B	B
Participant 2.3	D	D	D	A	E	E	A	B

Colored cells indicate a difference in preferred design.

#### 3.1.4 Correlation between criteria and prototype preference

The Spearman results suggest that explanations that are easy to use, concise, and satisfying tend to correlate with prototype preference. Notably, while *conciseness* was the most important factor in **Insurance Claims**, *satisfaction* was more strongly associated with **FinTech Credit**. This variation indicates that use-case specific needs influence how users perceive and evaluate explanations.

The correlation between *easy to use* and prototype preference in both use-cases suggests that explanations should prioritize usability to maximize their effectiveness. In contrast, attributes such as *trustworthy* and *useful* exhibit lower correlations; however, these results should be interpreted in light of the fact that the explanations were aligned with end-users' decision-making needs, and received relatively high ratings across all criteria. Because these attributes were already evaluated favorably—at or above the midpoint of the 5-point Likert scale—there was limited variation for them to exert a stronger influence on preference. Their lower correlations do not suggest that they are unimportant, but rather that, within an already sufficiently rated set of explanations, they are potentially less decisive than perceptions of ease of use.

Additionally, the absolute scores of each criterion for the preferred prototype designs indicate that they perform at a sufficient level as seen in Table 4. This finding suggests that these designs are not only preferred but also meaningful. This indicates that the designs align with user expectations and match the use-case.

### 3.2 Workshop results on generality of visual designs

The workshop provided insights into the strengths and weaknesses of the 20 explanation designs, covering four explanation types: *feature importance, contrastive/similar examples, counterfactuals, and rule-based explanations*. The evaluation followed a two-step mixed-method approach: first, participants ranked the prototypes using the *100-dollar method*, and after which they provided qualitative feedback on the top three designs per explanation type, focusing on usability and generalizability.

#### 3.2.1 100-dollar method

The results of the *100-dollar method* are presented in Table 7, with scores averaged across all participants. The findings indicate that Prototype D is the most preferred among participants in terms of Feature Importance. For counterfactual explanations, Prototypes C and E are the most favored ones. In the case of similar and contrastive examples, participants demonstrated a preference for Prototype A. Lastly, for rule-based explanations, Prototype B received the highest score. The rationale behind these preferences and the implications of the highest-rated prototypes are further analyzed in the subsequent sections.

**Table 7:** Ranking of explanation prototypes based on the 100-dollar method.

**Prototype**	**A**	**B**	**C**	**D**	**E**
Feature importance	32.5 ± 10.08	7.5 ± 7.68	2.5 ± 4.00	**47.5** ± 7.68	10 ± 6.56
Counterfactuals	27.5 ± 21.04	11.25 ± 5.04	**30** ± 6.56	**30** ± 28.49	1.25 ± 2.00
Similar/contrastive examples	**47.5** ± 13.68	2.5 ± 4.00	23.75 ± 11.92	8.75 ± 11.44	17.5 ± 13.68
Rule-based explanation	13.75 ± 14.40	31.25 ± 8.24	**32.5** ± 16.48	15 ± 13.84	7.5 ± 12.00

Higher scores indicate stronger preference among participants. The scores are averaged over participants and reported as mean ± 95% confidence interval. Bold values indicate the highest mean scores observed per explanation type.

#### 3.2.2 Feature importance explanations

Participants identified several challenges related to the interpretability and usability of feature importance visualizations. A recurring concern was the lack of clarity regarding the scale of values, as it was often unclear whether scores were normalized, what their maximum values were, or how they should be interpreted in context. Many participants also questioned how a system should handle cases with numerous features, debating whether to display only the most important ones or set a threshold for inclusion.

The use of color played a crucial role in interpretation, with red often signaling negative impact. However, some participants pointed out that the prototypes focused solely on highlighting potential risk factors, without presenting any mitigating information that could balance or contextualize those risks. This absence, they noted, may contribute to automation bias, as users might be more likely to accept negative system outputs without questioning or critically assessing them. Additionally, the effectiveness of color coding was debated, with some suggesting a green-to-red gradient to indicate varying levels of importance, while others were concerned that green might imply a beneficial impact even when a feature simply had a neutral or weak effect.

Design preferences varied regarding layout and information density. While some prototypes were criticized for excessive negative space, making them harder to interpret at a glance, others were praised for their compact and structured design.

#### 3.2.3 Contrastive/similar examples

Feedback on contrastive explanations largely revolved around readability and layout efficiency. Participants expressed a preference for a logical reading order, typically from left to right and top to bottom. Some prototypes disrupted this flow, requiring users to navigate back and forth, which made interpretation more cumbersome. Example positioning was also a point of discussion, with many emphasizing that the current case should be more prominently displayed, ideally in the top left or highlighted with additional visual effects.

The scalability of presenting multiple similar or contrastive examples emerged as a concern, particularly in prototypes that displayed several cases side by side. Participants questioned which examples should be selected and how many could feasibly be shown without overwhelming the user or diluting the relevance of the comparison. This raised broader issues around curating representative examples while maintaining clarity and usability. There was general interest in interactive elements, such as hover effects or selection mechanisms, to allow users to focus on specific examples without overwhelming them with information.

Design E featured a wave-like structure intended to represent a decision boundary between different possible outcomes of the model. However, during evaluation, the wave motif introduced unintended ambiguity. While the underlying model employed hard categorical decisions, wave-like shape implied probabilistic uncertainty or a soft boundary, which led to user confusion. This mismatch between visual metaphor and model behavior highlights the importance of aligning graphical representations with the actual semantics of the model. Furthermore, this feedback suggests that users tend to favor lean, unambiguous designs that do not introduce unnecessary visual complexity. This underscores that even subtle stylistic choices can affect user understanding in explanation interfaces.

#### 3.2.4 Counterfactual explanations

Reactions to counterfactual explanations were mixed, often influenced by the specific context in which they would be applied. While some participants appreciated the clean and structured nature of certain designs, others questioned whether counterfactuals were useful in all scenarios. The ability to visually compare original and modified cases was generally well received.

Color coding was again a topic of discussion, with some participants advocating for more intuitive risk indicators. In one prototype, for instance, a risk score was highlighted in red, even though the predicted risk level was medium. Several participants suggested that orange would be more appropriate in such cases, emphasizing the importance of selecting colors that semantically align with perceived risk levels.

Again, compact, lean layouts were preferred over repetitive or overly detailed displays, as excessive information made interpretation more difficult.

#### 3.2.5 Rule-based explanations

Decision rule visualizations were found to be highly context-dependent, making it difficult to determine a universally effective design. A decision tree can be simplified by collapsing it into a sequence of rules rather than depicting the full tree. We captured mixed preferences regarding the presentation of plain rules versus the whole decision tree.

Participants generally preferred designs with interactive elements, such as drop-down menus, which helped reduce visual clutter and supported intuitive navigation. Risk levels— high, medium, and low—were effectively communicated through familiar color shading (red, orange, green) and were widely understood. However, one prototype introduced a sequence of shaded boxes intended to represent a series of risk mitigation steps. Although the concept aimed to convey progression, participants found the visual flow ambiguous, making it difficult to discern the intended order or interpret meaning at a glance. This underscores the importance of clear visual hierarchies and cautions against overly abstract or non-standard design metaphors that may increase cognitive load. In contrast, participants who prioritized efficiency over detailed step-by-step representations preferred more compact, actionable, and straightforward designs, those that clearly highlight the outcome and suggest how risks might be mitigated.

#### 3.2.6 Preliminary design principles

This section presents context-specific insights derived from user studies and workshop discussions conducted throughout the design process. Given the limited scope of two use cases in a single industry and a total of six end-users, these findings should be interpreted as exploratory and illustrative.

##### 3.2.6.1 Use of color

Users tend to associate red with negative and green with positive outcomes, due in part to common real-world associations such as traffic signals or software indicators. While we used red to mark negative outcomes, leveraging its strong intuitive signal, we intentionally avoided using green for positive outcomes to reduce the risk of overly strong or biased interpretations. Instead, we chose light blue as a more neutral alternative.

This choice reflects the nature of the decision-making context in our studies. The values being visualized are not inherently “good” or “bad” but represent a continuum of approval levels. A lower score, for instance, might simply indicate a lesser degree of risk rather than a definitively positive outcome. Blue was found to be a more neutral and appropriate color for such nuanced interpretations. Importantly, this also preserves the use of green for truly positive confirmations.

##### 3.2.6.2 Contextual information

Many data visualizations rely solely on icons, numbers, or simplified graphics, which often strip away essential context. Users reported that visualizations lacking explanatory information—such as bar charts without numeric values or raw scores without interpretation—felt incomplete or even confusing. To improve comprehension, we added visual “hints” such as numeric breakdowns and comparative scales.

Users appreciated having just enough information to understand a result without feeling overwhelmed by data they could not immediately interpret.

##### 3.2.6.3 Visual restraint

Effective visual design requires restraint. Not every data point needs to be visualized, and minimalism can often be more informative than information overload. Users process visuals faster than text, so visual cues—such as shapes, icons, or layout—were used strategically to communicate key insights at a glance.

In our designs, we focused on including only the elements necessary for a meaningful understanding of an outcome. For example, rather than showing raw scores alone, we contextualized them with relevant reference points and brief explanations.

##### 3.2.6.4 Structure and visual hierarchy

Clarity also depends on how information is arranged. Realistic, freeform layouts may be visually rich, but they often lack the structure needed for efficient comprehension. Instead, we emphasized ordered layouts, logical grouping of elements, and visual hierarchy to guide the user's eye.

Typography size, contrast, and positioning were leveraged to indicate importance and encourage a left-to-right, top-down reading pattern, aligned with Western reading habits. This balance aims to reduce cognitive load while still directing attention where it mattered most.

##### 3.2.6.5 Cognitive load

The cognitive load imposed by certain visualizations was also a key consideration. Some designs, particularly in the contrastive and counterfactual categories, presented information in ways that were visually appealing but cognitively demanding. Participants often preferred more compact, structured layouts over those that relied on excessive whitespace or overly complex designs. Additionally, the ordering of elements seem to influence usability, with a strong preference for intuitive reading flows,

##### 3.2.6.6 Generalizability

A major challenge in XAI visualization design is ensuring generalizability across different use-cases. Many stakeholders, particularly consultancy partners, emphasized that explanation visualizations need to be adaptable to various domains and decision-making contexts. In some cases, end-users needed to make quick decisions based on explanations, favoring concise and direct visualizations. In other cases, users required more nuanced information to balance competing factors, necessitating richer, interactive designs.

This variability in user needs might indicate that a single, one-size-fits-all explanation is unlikely to be effective. Instead, flexible, customizable explanations that allow users to adjust the level of detail or interact with different elements may provide a better balance between usability and comprehensiveness. Additionally, interactive features such as hover effects, tooltips, or filtering options could help tailor explanations to different levels of expertise and situational demands.

## 4 Discussion

This study highlights the potential role of human-centered design in Explainable AI (XAI) for financial decision-making. While based on a limited sample size, our findings suggest that the perceived meaningfulness of AI explanations is influenced by design choices, such as layout and color coding. Participants tended to prefer explanations that were easy to use, satisfying, and—depending on the context—concise. Furthermore, the results indicate that different explanation formats may serve distinct user needs, underscoring the importance of adaptable and context-sensitive designs. These findings should be interpreted as exploratory and motivate further validation with larger and more diverse user populations.

A key strength of this research is its mixed-method approach, which combines a user study with end users and a workshop with other stakeholders. This allowed us to capture both end-user preferences and broader regulatory and professional perspectives, ensuring the relevance of our findings across multiple stakeholders. Additionally, this study contributes empirical evidence on how XAI design choices influence usability and how this impacts the quality of explanations experienced by its users—an area often overlooked in more technically focused XAI research.

Our findings revealed a notable discrepancy between end-users' preferences and those of other key stakeholders regarding explanation design. This divergence is particularly evident in the case of rule-based explanations. While users favored simpler, more compact representations—such as minimal sets of rules that support interpretability and reduce cognitive load—stakeholders including XAI consultants, compliance officers, and developers expressed a preference for more complete representations, such as full decision trees that capture the entirety of the model's logic. This tension highlights a trade-off between interpretability and completeness that is contingent on the audience's needs and goals.

A similar divergence was observed in the use of similar and contrastive example-based explanations. End-users expressed a preference for visualizations, particularly graphical risk plots, which helped them contextualize predictions relative to similar or contrasting cases. In contrast, other stakeholders prioritized concise, structured tabular formats.

These differences underscore the importance of tailoring explanation strategies not only to users but also to the broader ecosystem of stakeholders involved in the deployment and oversight of AI systems. A one-size-fits-all approach to explainability may fall short; instead, layered or customizable explanations may better accommodate varying expectations and information needs across stakeholder groups.

Despite these strengths, the study has limitations. The sample size was small and drawn from two financial organizations, which may limit generalizability; broader and more diverse samples across domains would strengthen future work. Additionally, the focus on financial decision-making may reduce applicability to other high-risk domains such as healthcare or emergency response. Finally, real-world deployment and longitudinal studies are needed to evaluate long-term effects on decision-making and trust in AI.

Similar to prior studies, we found that users have difficulty with ambiguous color schemes and unclear scoring metrics (Doshi-Velez and Kim, 2017). Furthermore, our study extends existing literature by providing empirical rankings of explanation prototypes, reinforcing the argument that design elements impact user comprehension and trust (Mohseni et al., 2021). Importantly, this study confirms that the effectiveness of XAI explanations depends not only on their transparency but also on their ability to support efficient decision-making (Kaur et al., 2020).

Future research should explore XAI design in real-world settings to assess how explanations influence long-term trust and user behavior. Notably, for some explanation types, multiple designs were highly ranked, suggesting that no single approach fits all users or contexts. This points to the need for future studies to explore adaptive and personalized explanation mechanisms that can adjust based on user expertise, organizational and situational context. Effective XAI requires integrating technical accuracy with human-centered design principles, while considering the cognitive factors affecting user engagement. Another direction is the exploration of interactive explanations, where users can dynamically engage with AI outputs to refine their understanding. Bridging the gap between algorithmic transparency and usability demands interdisciplinary collaborations between AI researchers, cognitive scientists, and UX designers, which will be essential to developing more effective and human-centered XAI solutions that support informed decision-making.

## Data Availability

The datasets presented in this study can be found in online repositories. The names of the repository/repositories and accession number(s) can be found in the article/Supplementary material.
