# Supplementary material for: Designing effective explainable AI: a human-centered evaluation of explanation formats in financial decision-making
Source: Front Artif Intell. 2026 Mar 5;9:1668029. doi: 10.3389/frai.2026.1668029 (PMC12999942; doi:10.3389/frai.2026.1668029)
Supplement: Supplementary file 1 [file Data_Sheet_1.zip › supplementary/supplementary.pdf]

frontiers

Supplementary Material

1 SUPPLEMENTARY FIGURES AND TABLES

1.1 Figures

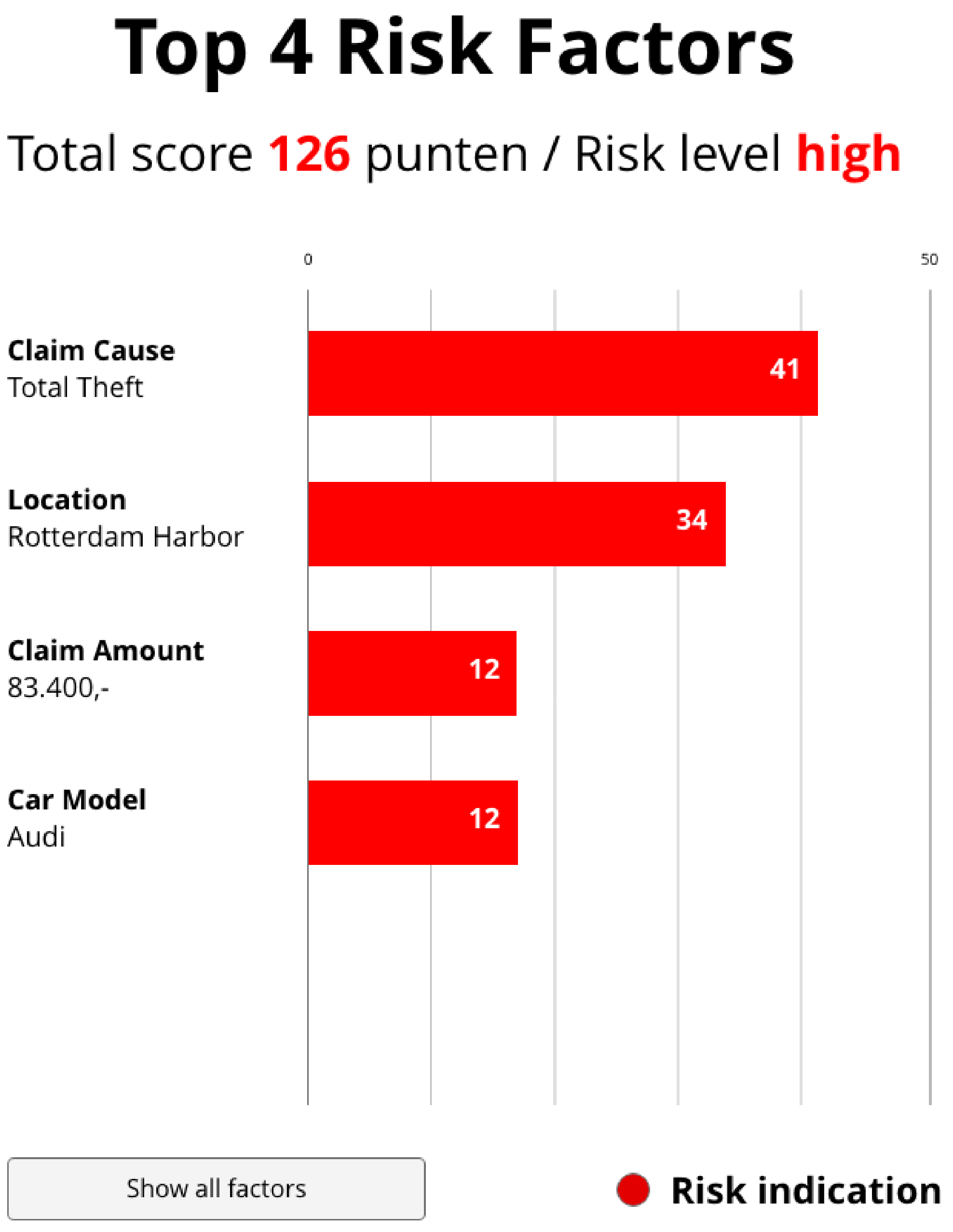

Figure S1. Feature Importance Design A

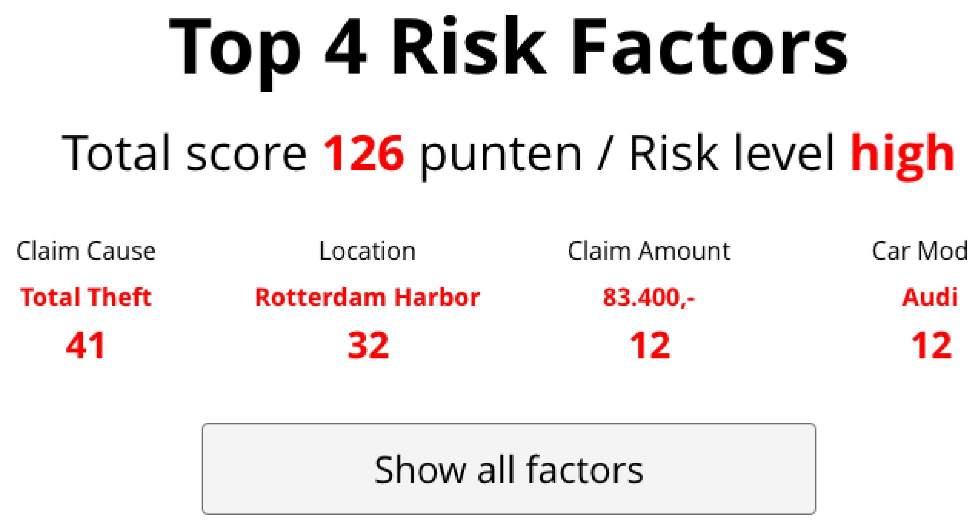

Figure S2. Feature Importance Design B

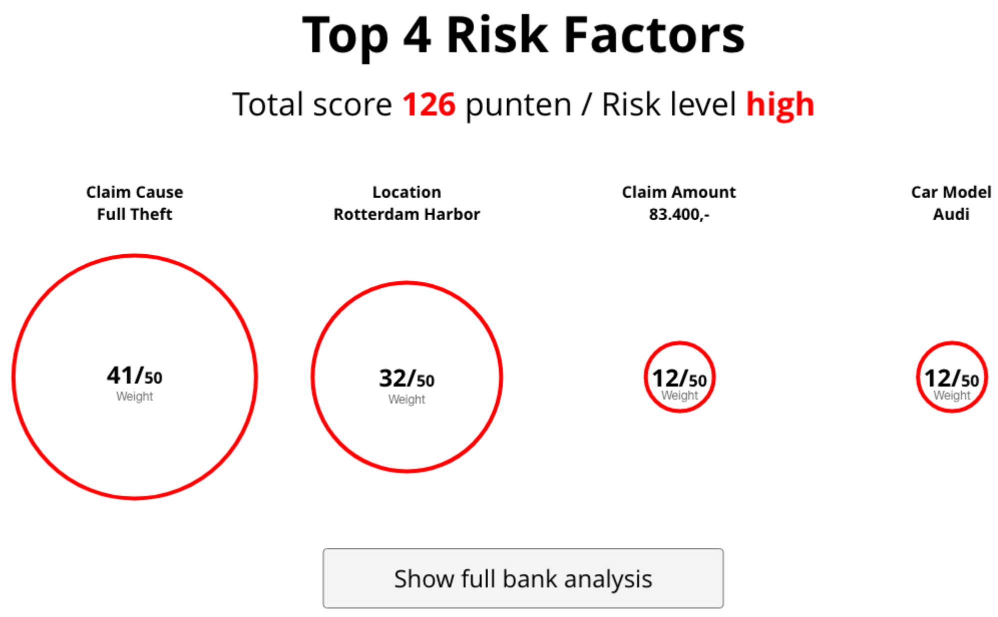

Figure S3. Feature Importance Design C

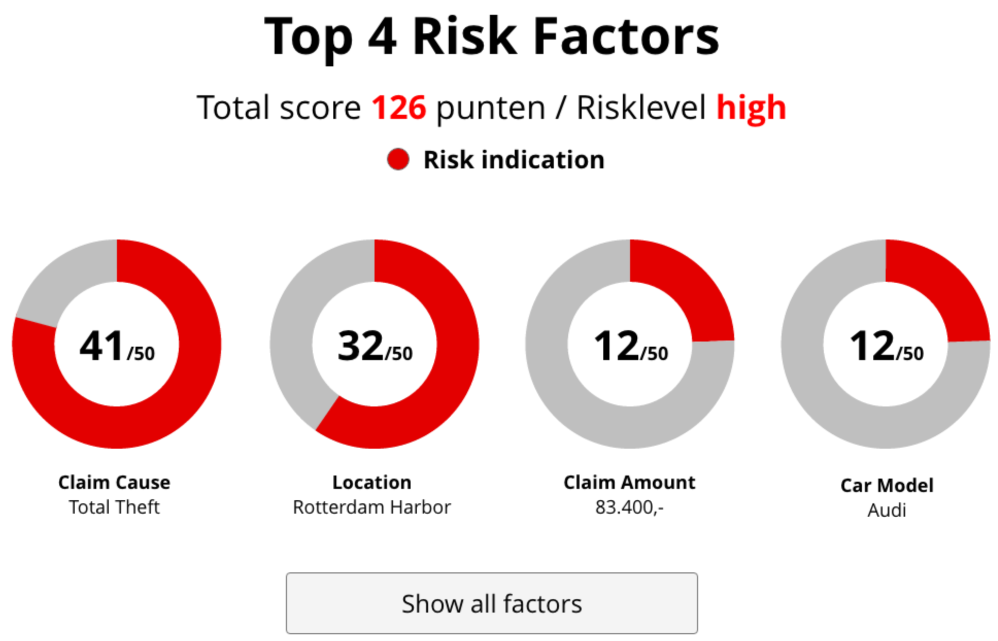

Figure S4. Feature Importance Design D

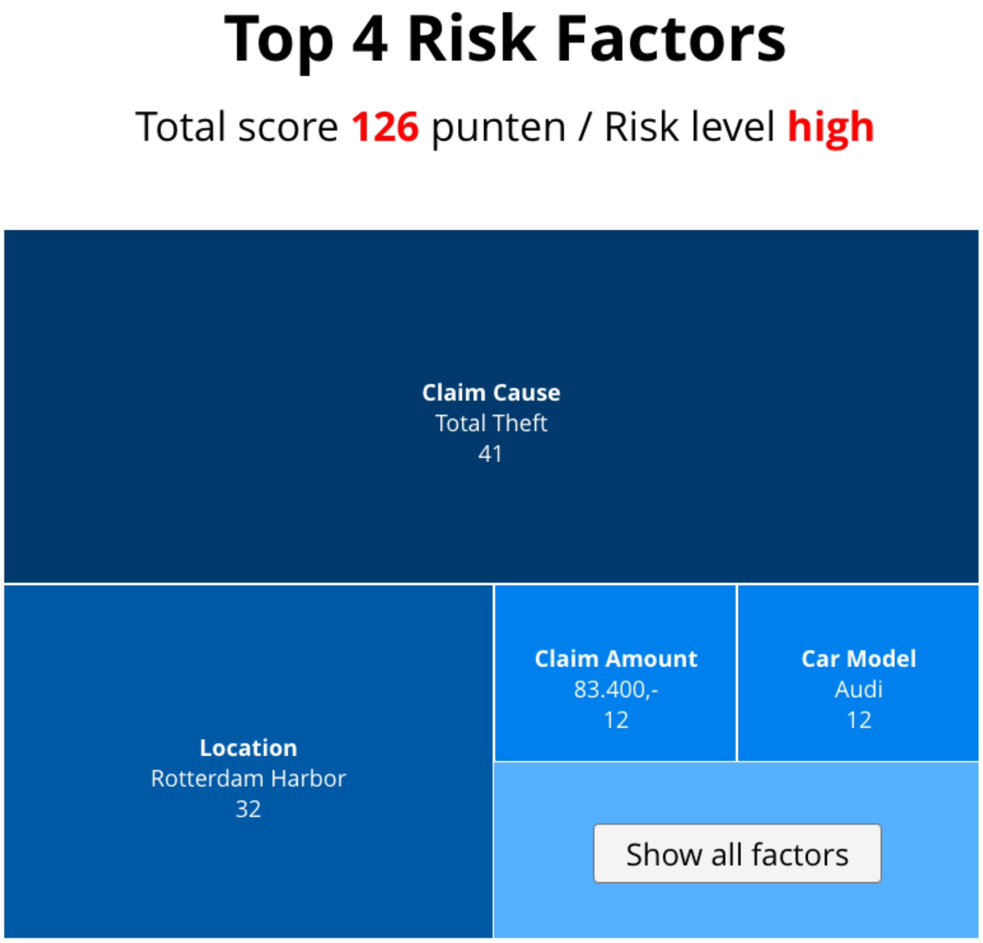

Figure S5. Feature Importance Design E

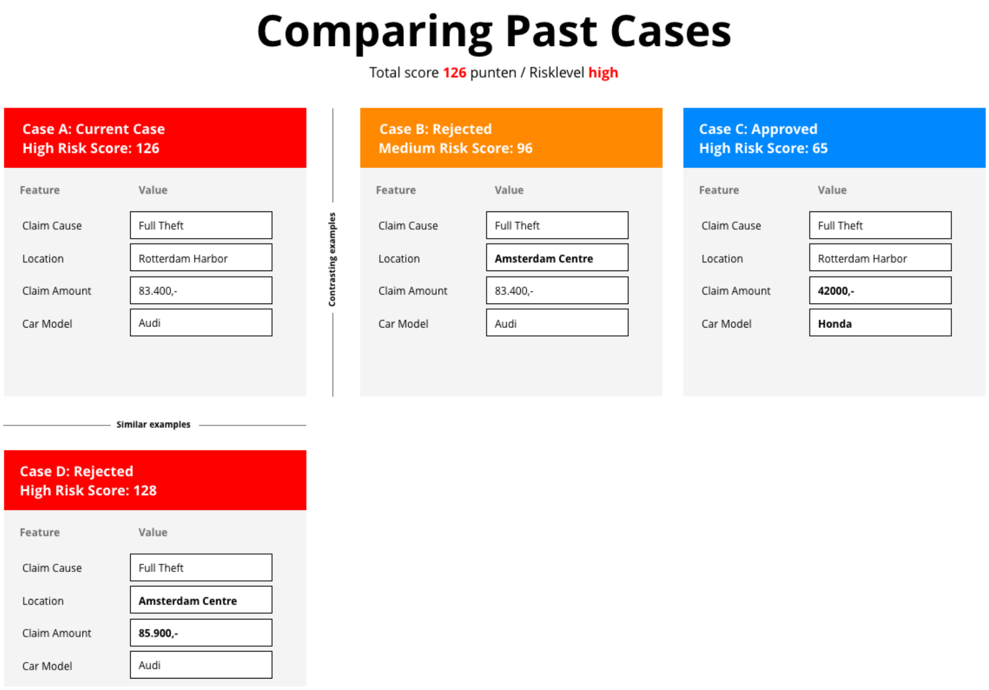

Figure S6. Contrastive / Similar Examples Design A

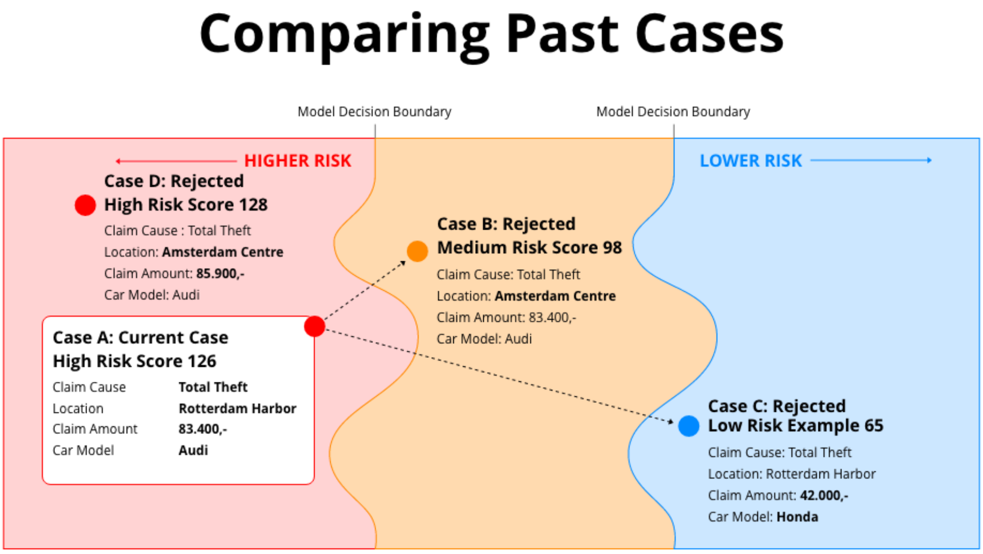

Figure S7. Contrastive / Similar Examples Design B

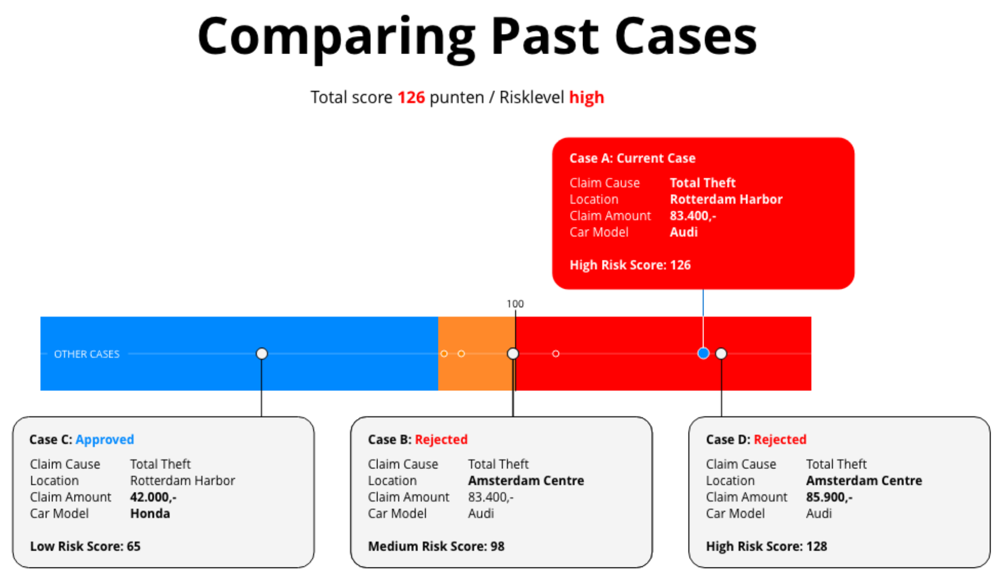

Figure S8. Contrastive / Similar Examples Design C

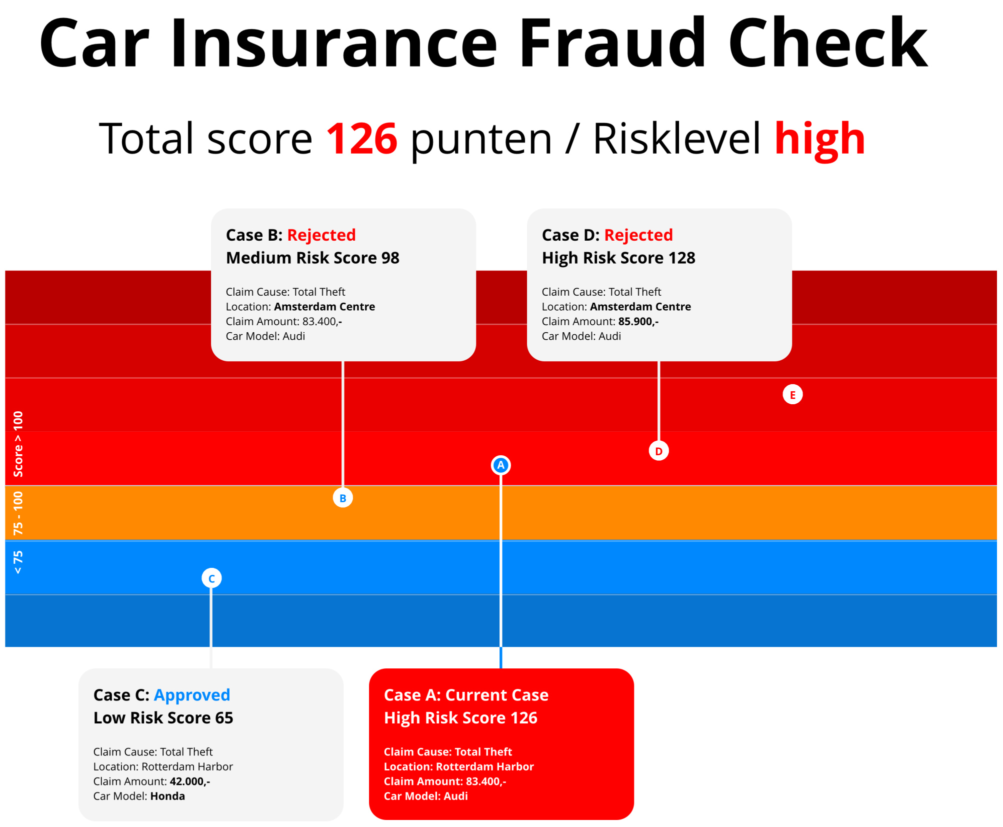

Figure S9. Contrastive / Similar Examples Design D

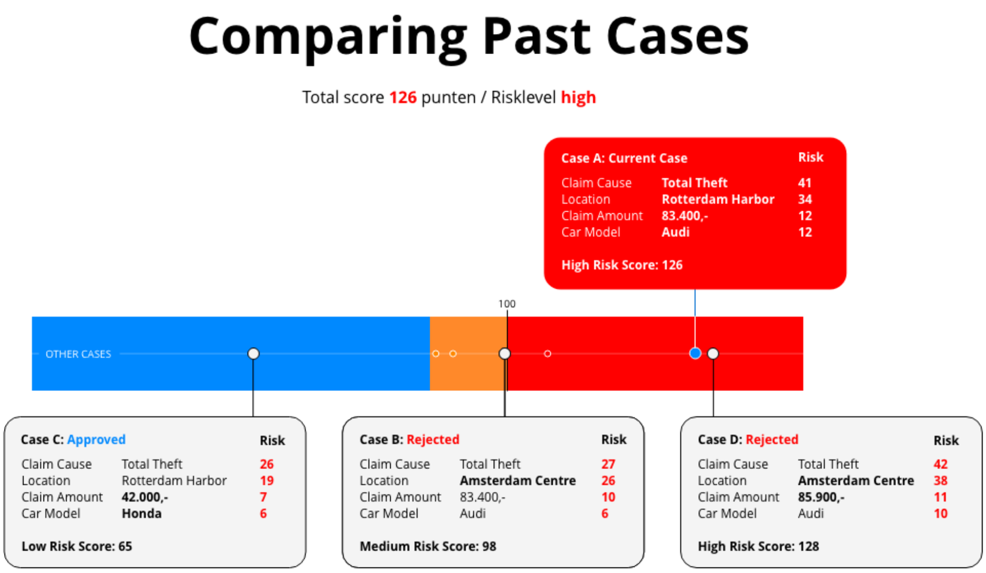

Figure S10. Contrastive / Similar Examples Design E

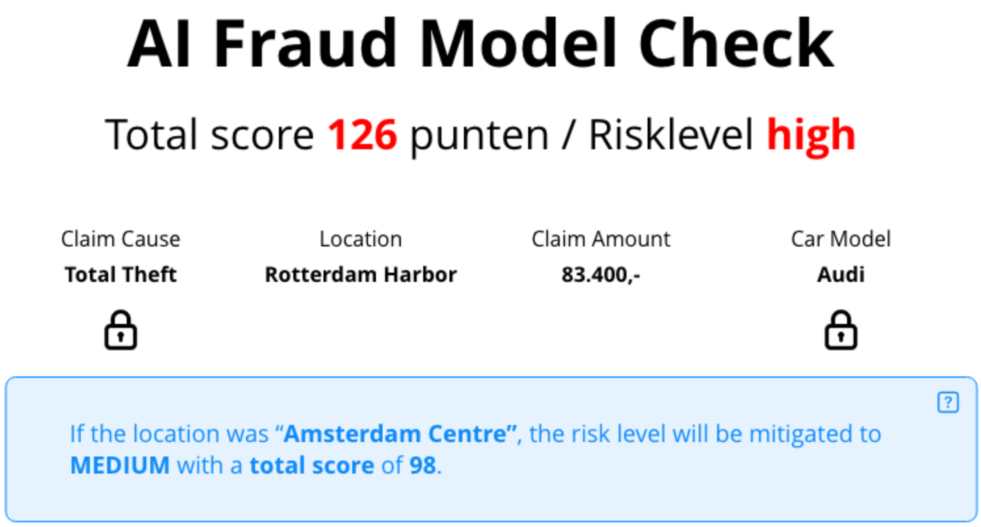

Figure S11. Counterfactuals Design A

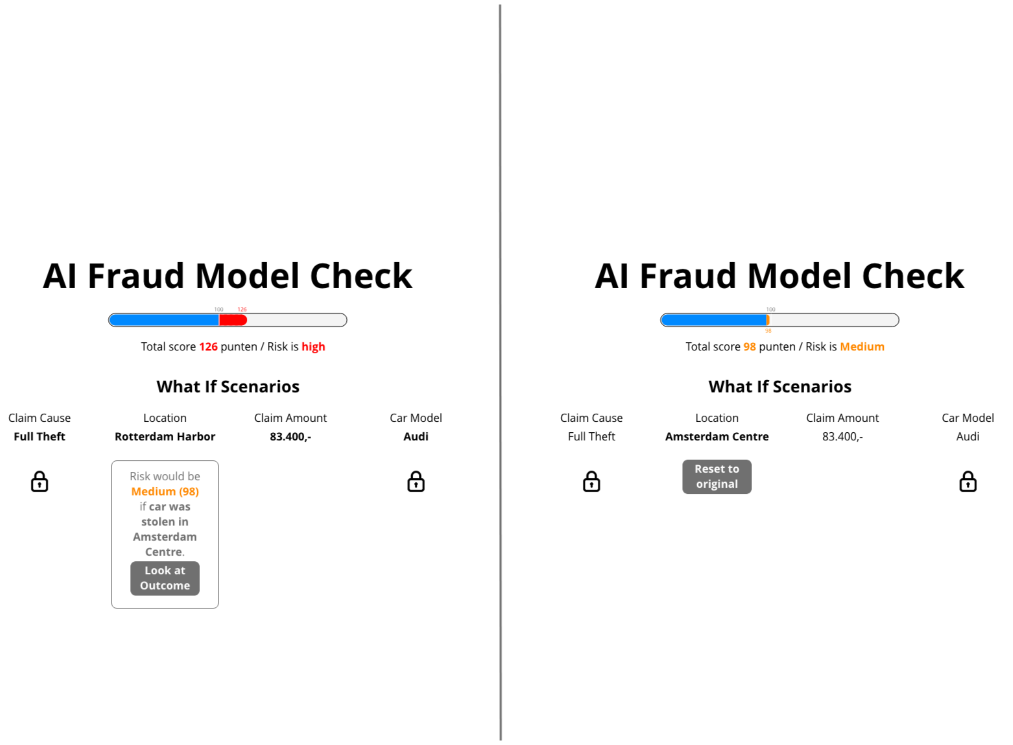

Figure S12. Counterfactuals Design B

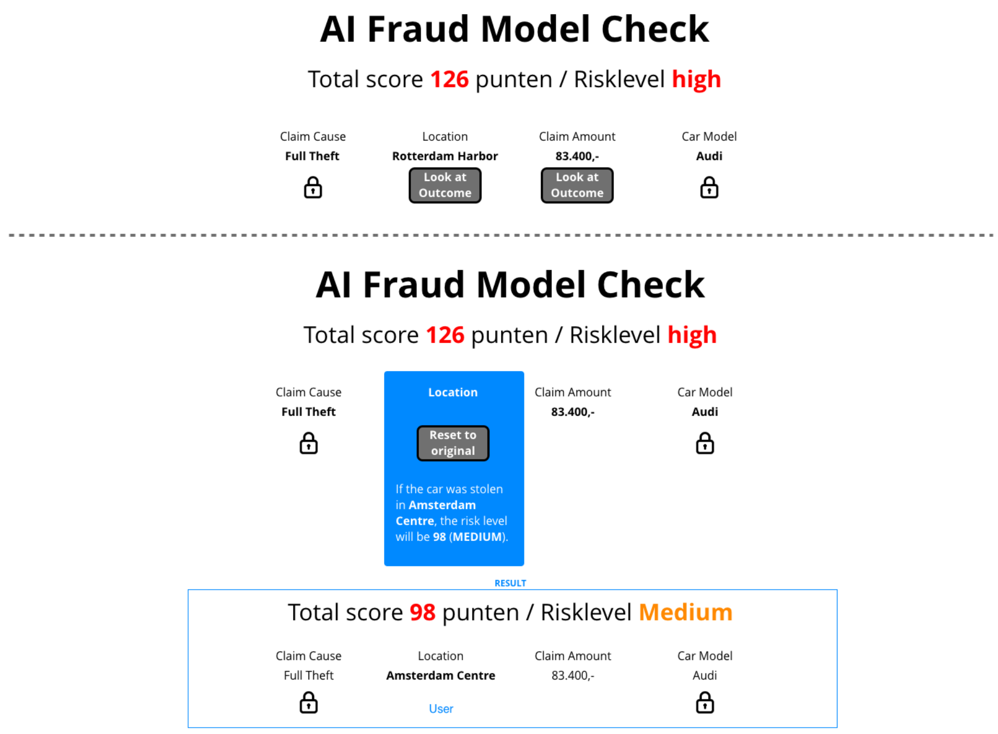

Figure S13. Counterfactuals Design C

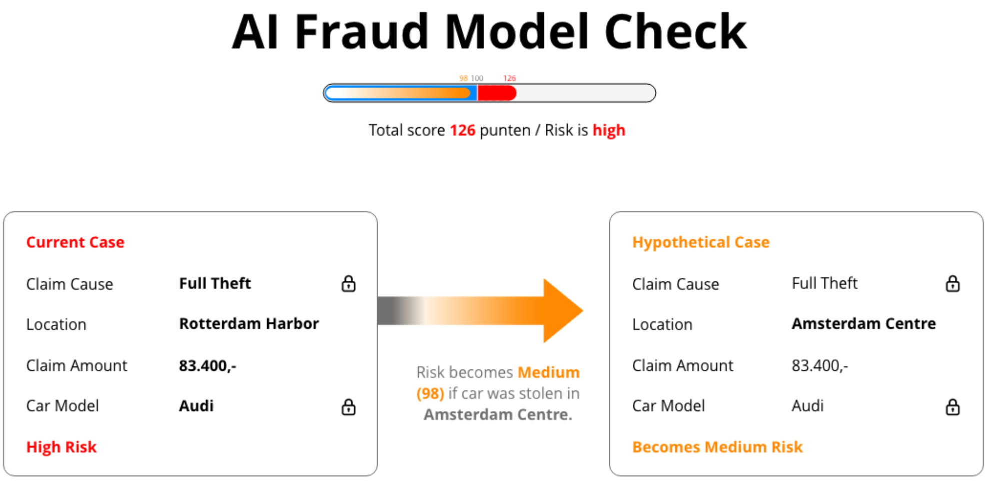

Figure S14. Counterfactuals Design D

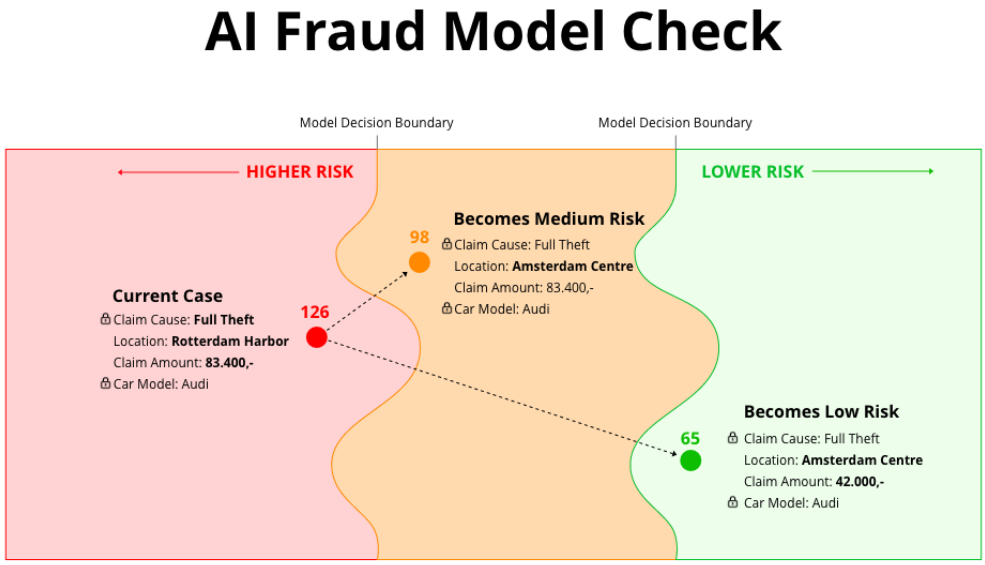

Figure S15. Counterfactuals Design E

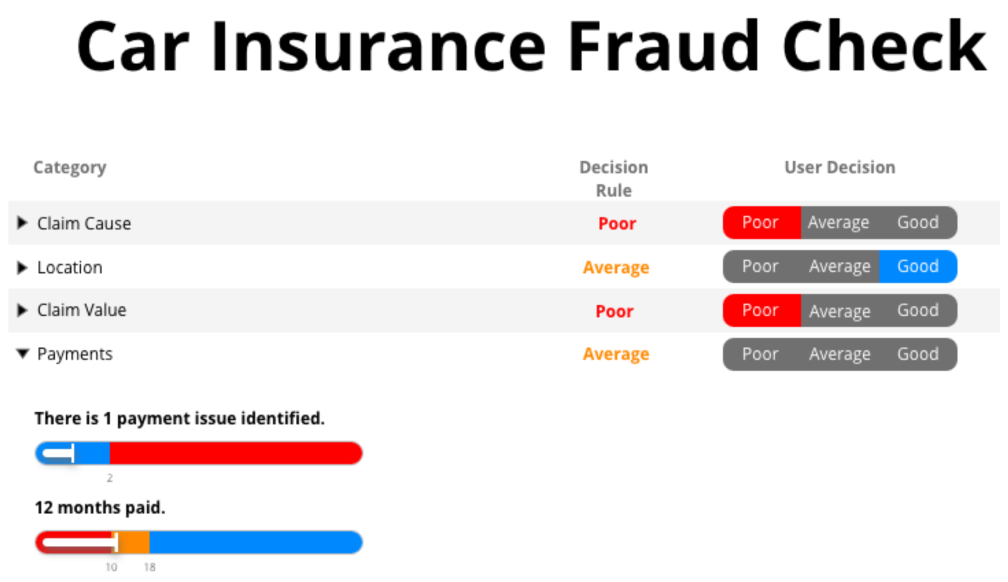

Figure S16. Rule-Based Explanations Design A

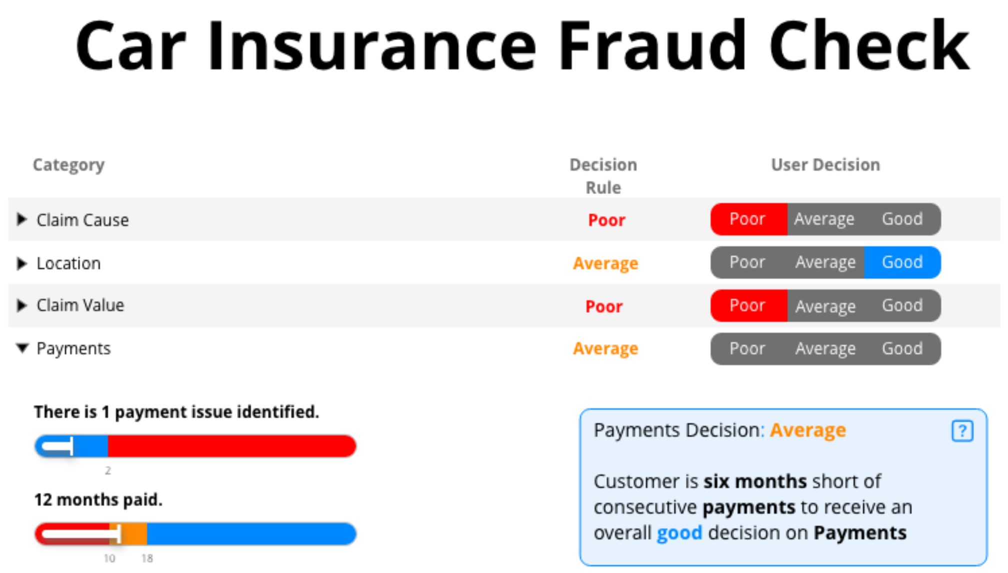

Figure S17. Rule-Based Explanations Design B

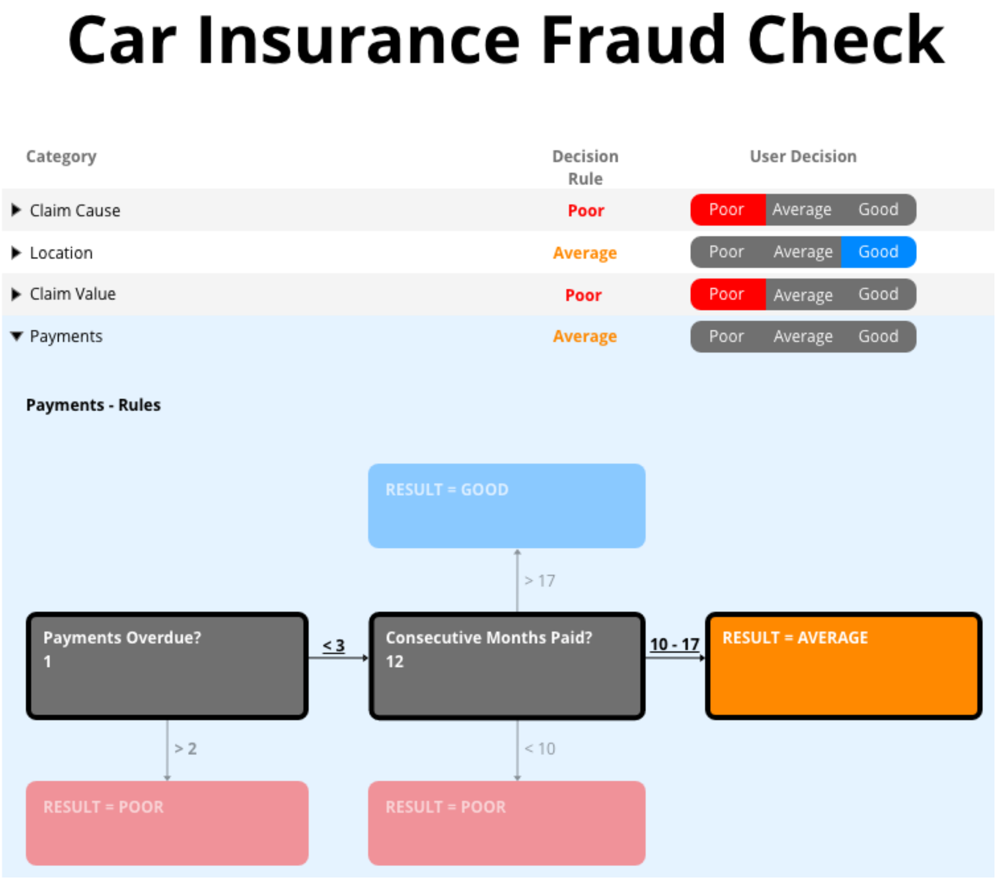

Figure S18. Rule-Based Explanations Design C

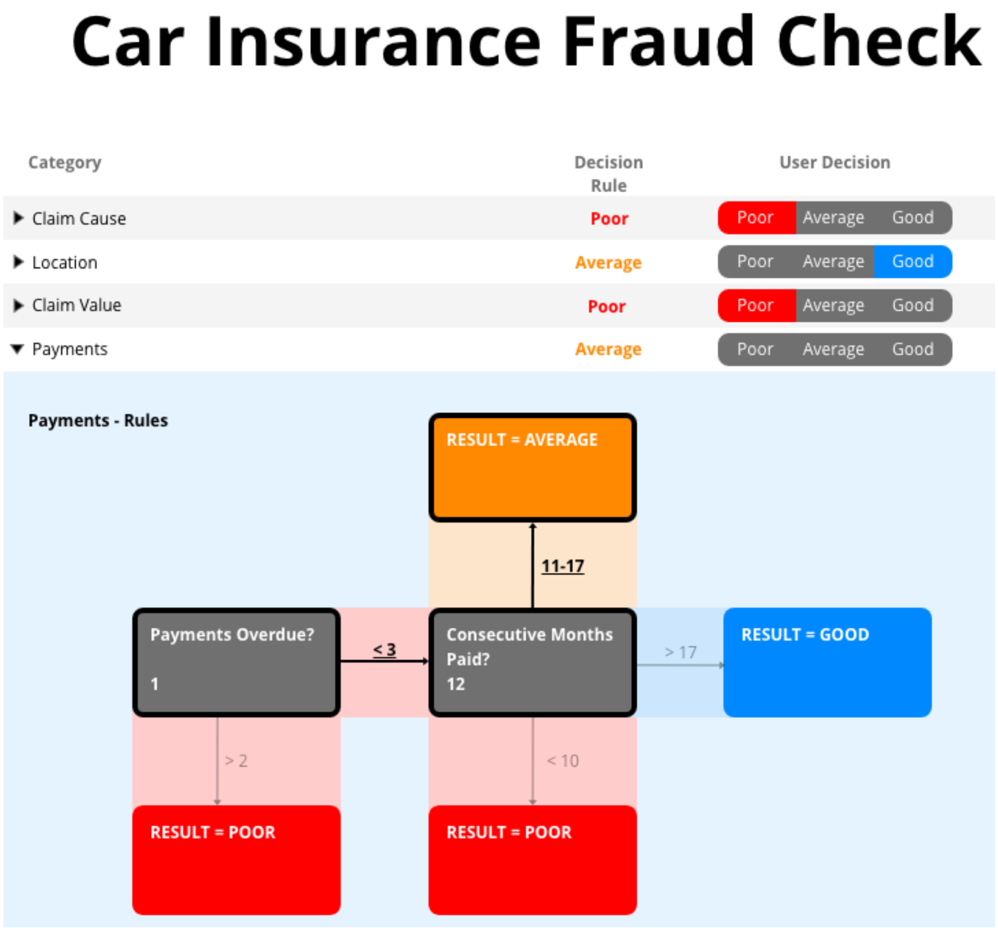

Figure S19. Rule-Based Explanations Design D

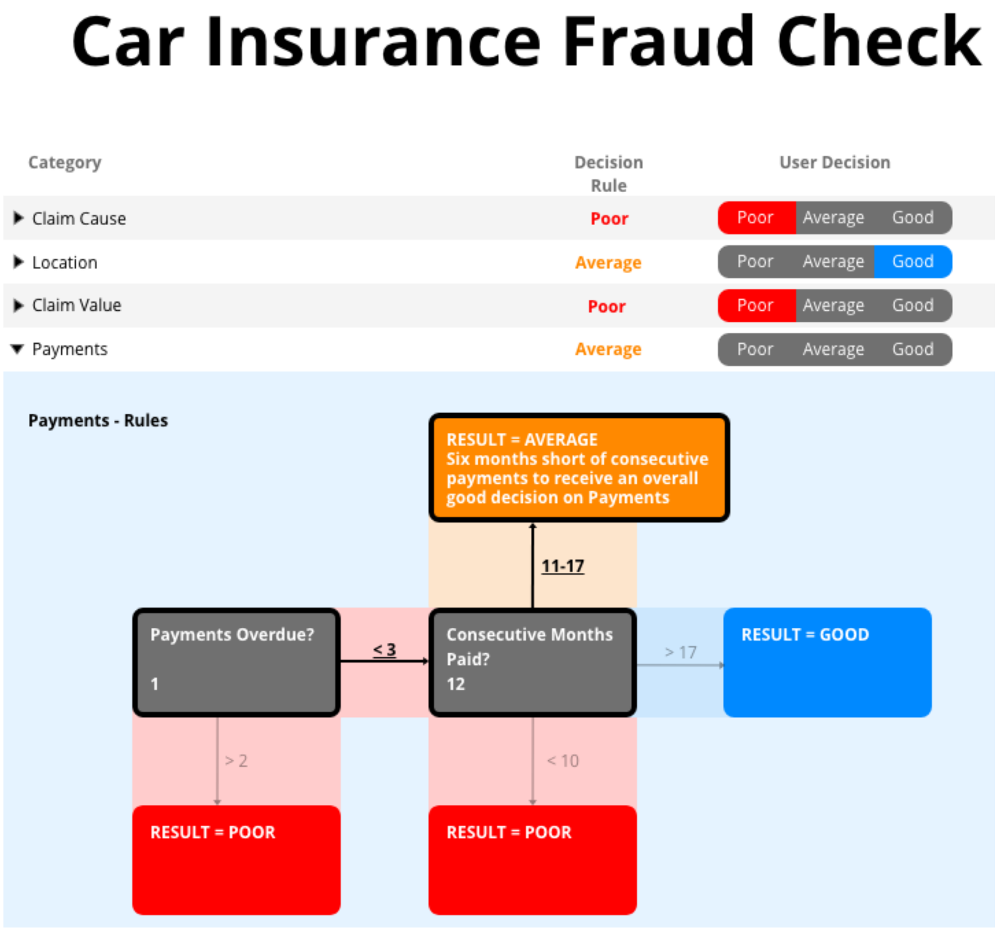

Figure S20. Rule-Based Explanations Design E

1.2 Tables

#	**Question**
1	Name of the use case (short description)
2	As complete a description of the use case as possible.<br>• What decision-supporting systems are used?<br>• Are these AI (Artificial Intelligence (AI), Machine Learning (ML) and/or Business Rules (BR) systems?<br>• If AI/ML systems are used, what type of AI/ML is applied (e.g., logistic regression, XGBoost, rule-based, neural network, etc.)?<br>• Since when have the decision-support systems been in use?<br>• What is the task or function of the decision-support systems?<br>• What are the inputs and outputs (data) of the systems?<br>• What processes are supported by the systems, and what steps are identified within those processes?<br>• Who are the stakeholders of these systems, and what are their roles (e.g., customers, regulators, auditors, etc.)?
3	What type of internal users receive the outcomes of the decision-supporting systems, and what do they do with them?
4	Do these internal users also receive explanations about the outcomes of the systems?<br>• If so:<br>• How is the outcome presented to users?<br>• What explanation is provided?<br>• How is the explanation communicated to internal users?<br>• How is the explanation generated (what kind of technique is used)?
5	To what extent is there a need for explanation in this use case for internal users?
6	Is there a policy within the organization regarding explainability or explainable AI?<br>• If so, what does that policy state?

Table S1: Questionnaire for Eliciting Use Case Information.

2 INTERVIEW PROTOCOL: INTERNAL USERS OF USE CASE(S)

Purpose

This interview protocol is intended for internal users of use case(s).

Introduction

Artificial Intelligence (AI) has been widely used in the financial sector in recent years. In particular, automated decision-making systems have become an essential tool for processes such as customer acceptance and transaction monitoring, enabling more efficient, effective, and consistent decision-making.

The purpose of this interview is to gather valuable insights about the use case, which will be used to generate meaningful explanations for your specific application of automated decision-making systems. We would like to understand the specific challenges and opportunities that arise from using such systems in your role, how Explainable AI can help improve transparency and accountability, and how the risks associated with using AI systems are minimized.

To collect this information, we have prepared a set of questions related to your role in the process.

Informed Consent Procedure

1. Ask whether the interviewee has read the information letter.
    - If yes: proceed to the next step.
    - If no: provide the information letter to the interviewee.
2. Hand over the informed consent form to the interviewee for reading and signing.
    - If the form is signed by both the interviewer and the interviewee: proceed to the next step.
    - If not: stop the interview.

Demographic Information

- Briefly describe your current role within the organization.
- How long have you been working in this role and in the industry in general?
- Have you been involved in the implementation of the decision support systems in the organization? If so, what role did you play?

Interview Questions: Internal Users

User Experience

The interviewer indicates that the focus is on the process involved in using the decision support system. If possible, the internal user guides the interviewer through the process on a screen.

If the process can be followed:

- Demonstrate the process from start to finish for a particular case.
- What steps do you take in each phase?
- What are you trying to accomplish in each step?

- How do you perform the step?
- What problems do you encounter, and how do you deal with them?
- What additional information or functionalities could improve the system's comprehensibility (transparency, explainability)?

If the process cannot be followed:

- Could you describe a recent task where you used the decision support system?
- What role did the system play in that task?
- What problems do you encounter, and how do you deal with them?
- What features or functionalities could enhance the system's comprehensibility?

Taking Decisions

- What do you consider the most important features or variables when making a decision?
- What is the impact of a decision on the customer? What information do they receive and how?
- How do you make a decision if no explanation is available?
- What are the most important criteria for you in decision-making?
- In which situations do you deviate from the system's outcomes, and why?
- Have you ever changed a decision based on an explanation from the system?
- What constitutes a good explanation from the system in your opinion?
- What are some hard cases you have encountered?
- Do you have any concerns or reservations about using the system for decision-making?
- How do you perceive your level of control over the decision support system?

Wrapping Up

- Kindly summarize the most important points that we discussed.
- Do you have any questions, comments, or additions based on this interview?

Thank you kindly for your cooperation!

3 QUESTIONNAIRE EVALUATING PROTOTYPES

Statement	Strongly Disagree	Disagree	Neutral	Agree	Strongly Agree
I understand the explanation	☐	☐	☐	☐	☐
The explanation is easy to understand	☐	☐	☐	☐	☐
The explanation is easy to use	☐	☐	☐	☐	☐
The explanation is satisfying	☐	☐	☐	☐	☐
The explanation is useful to achieve my goals	☐	☐	☐	☐	☐
The explanation is trustworthy and reliable	☐	☐	☐	☐	☐
The explanation is typical for similar cases, AI outputs or decisions	☐	☐	☐	☐	☐
The explanation has sufficient detail	☐	☐	☐	☐	☐
The explanation seems correct	☐	☐	☐	☐	☐
The explanation is concise and not too detailed	☐	☐	☐	☐	☐
The explanation tells me how to act next	☐	☐	☐	☐	☐
