# Supplementary figures and images for: Designing effective explainable AI: a human-centered evaluation of explanation formats in financial decision-making
Source: Front Artif Intell. 2026 Mar 5;9:1668029. doi: 10.3389/frai.2026.1668029 (PMC12999942; doi:10.3389/frai.2026.1668029)

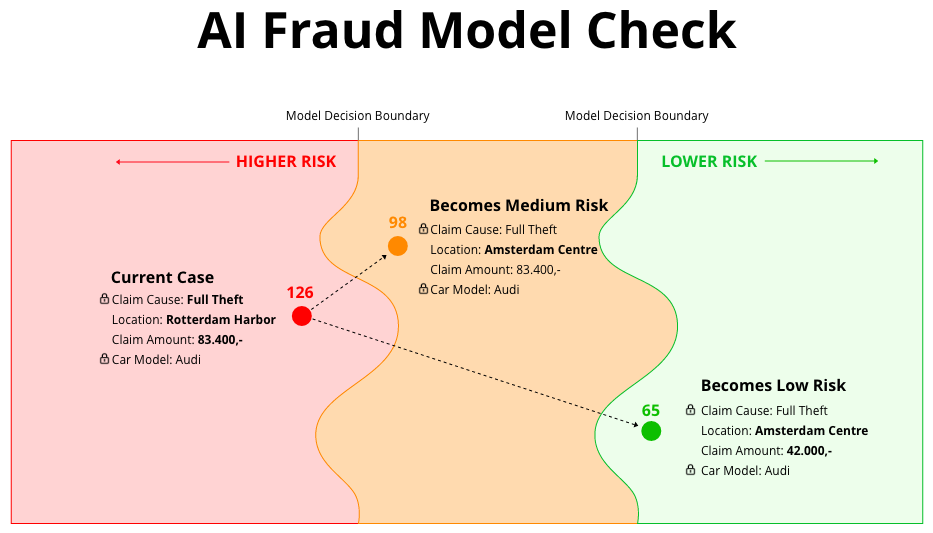

Supplement: Supplementary file 1 [file Data_Sheet_1.zip › supplementary/cropped_cf_4.jpg]

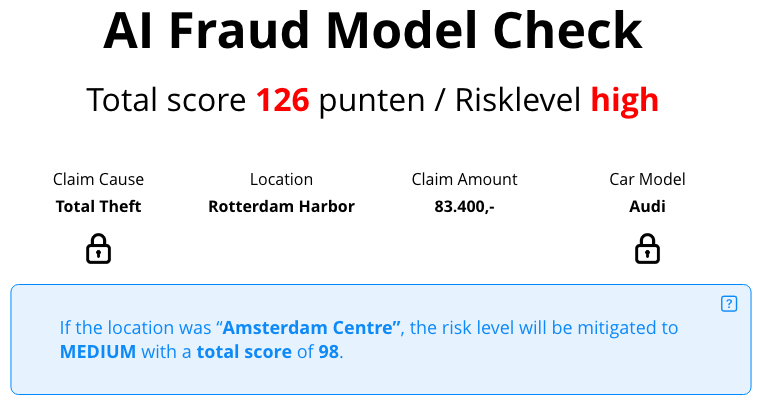

Supplement: Supplementary file 1 [file Data_Sheet_1.zip › supplementary/cropped_cf_0.jpg]

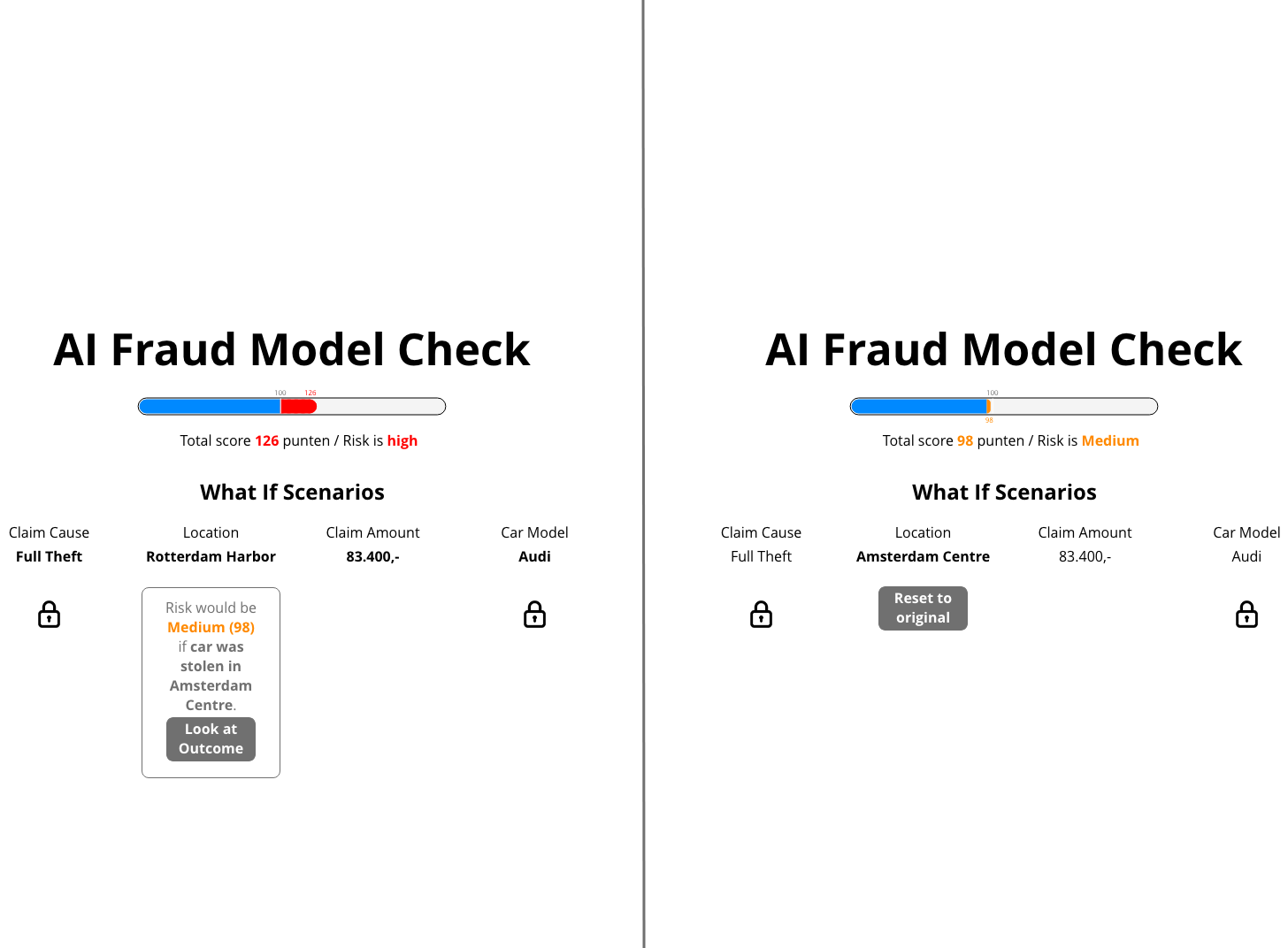

Supplement: Supplementary file 1 [file Data_Sheet_1.zip › supplementary/cropped_cf_1.jpg]

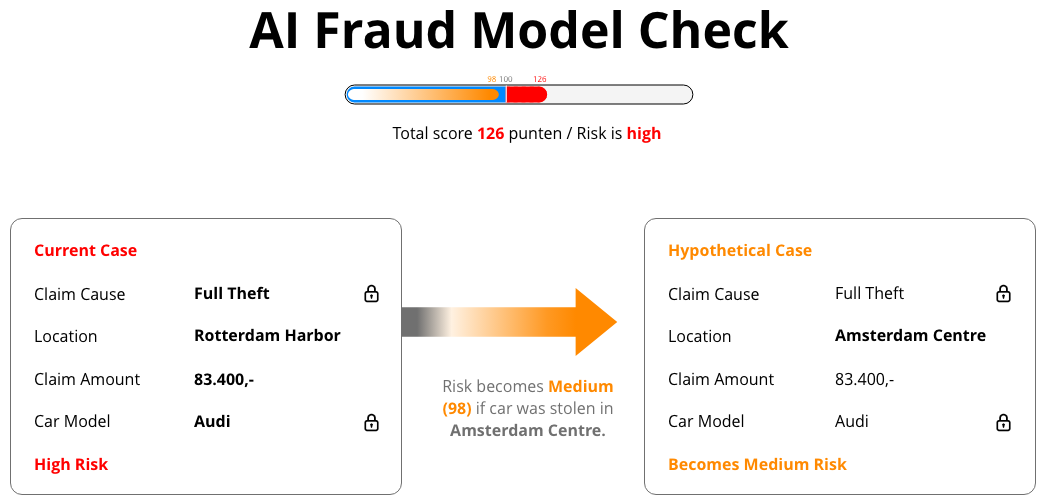

Supplement: Supplementary file 1 [file Data_Sheet_1.zip › supplementary/cropped_cf_3.jpg]

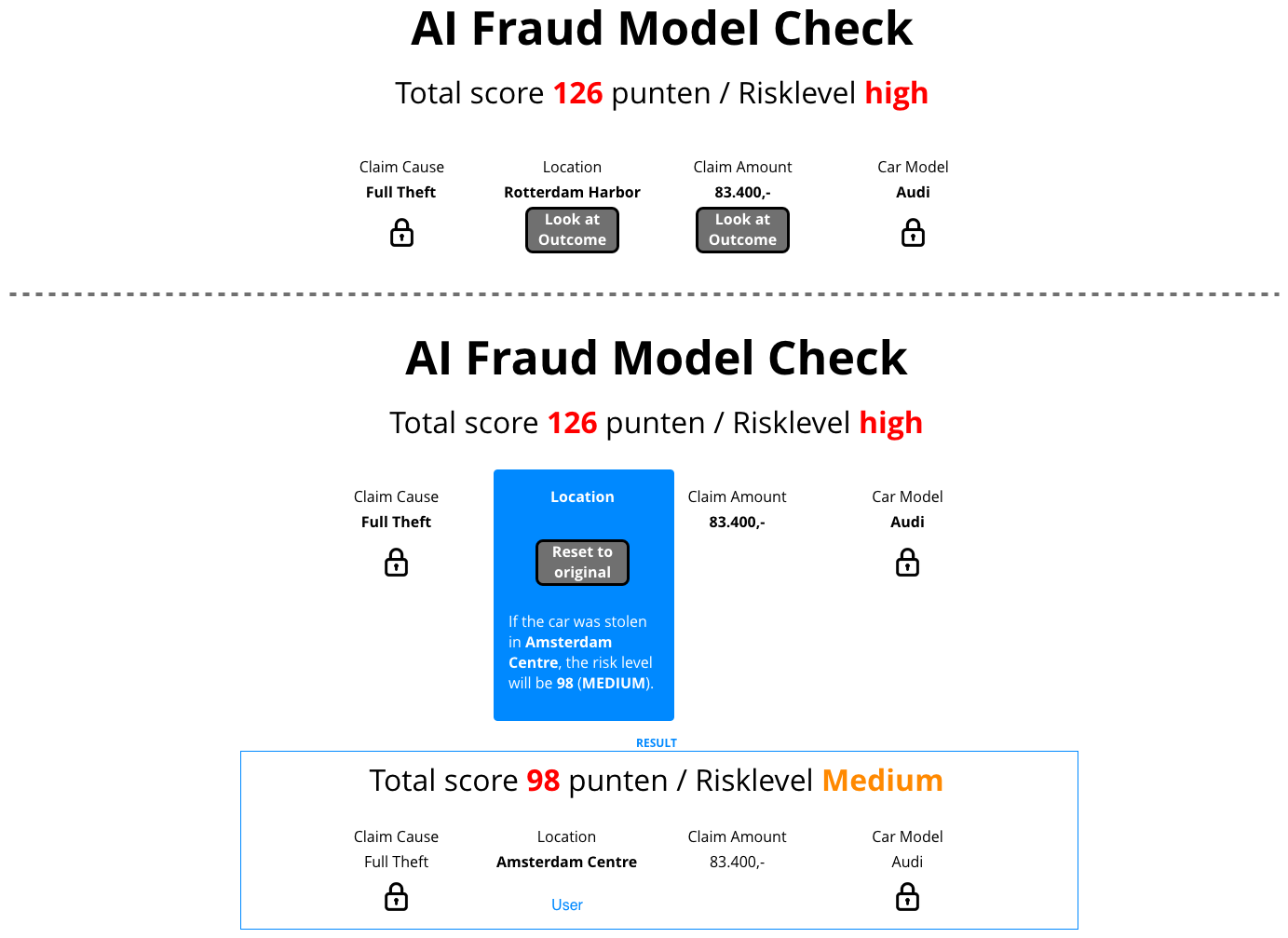

Supplement: Supplementary file 1 [file Data_Sheet_1.zip › supplementary/cropped_cf_2.jpg]

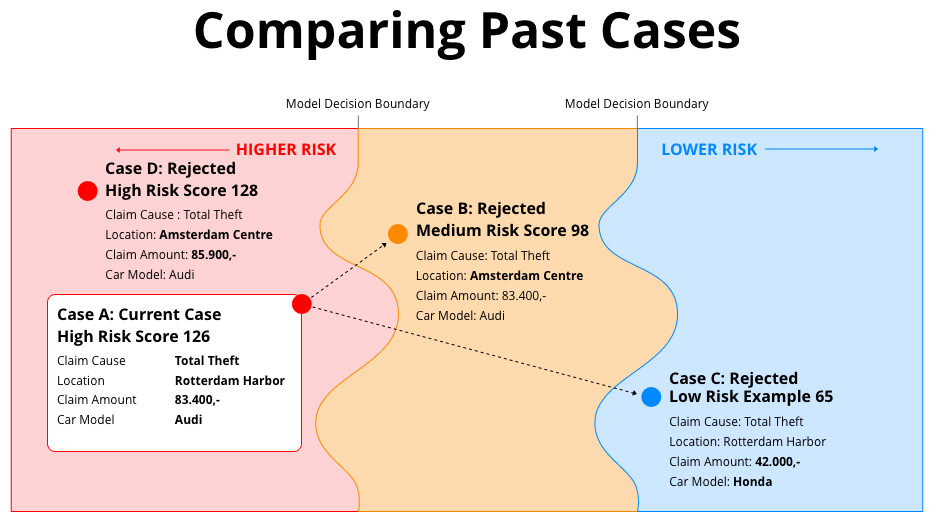

Supplement: Supplementary file 1 [file Data_Sheet_1.zip › supplementary/cropped_contrastive_1.jpg]

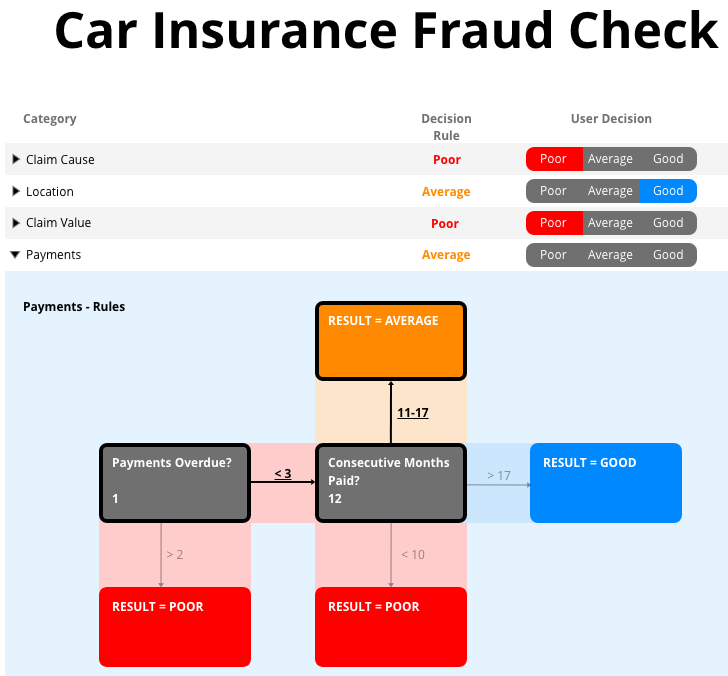

Supplement: Supplementary file 1 [file Data_Sheet_1.zip › supplementary/cropped_beslisregels_3.jpg]

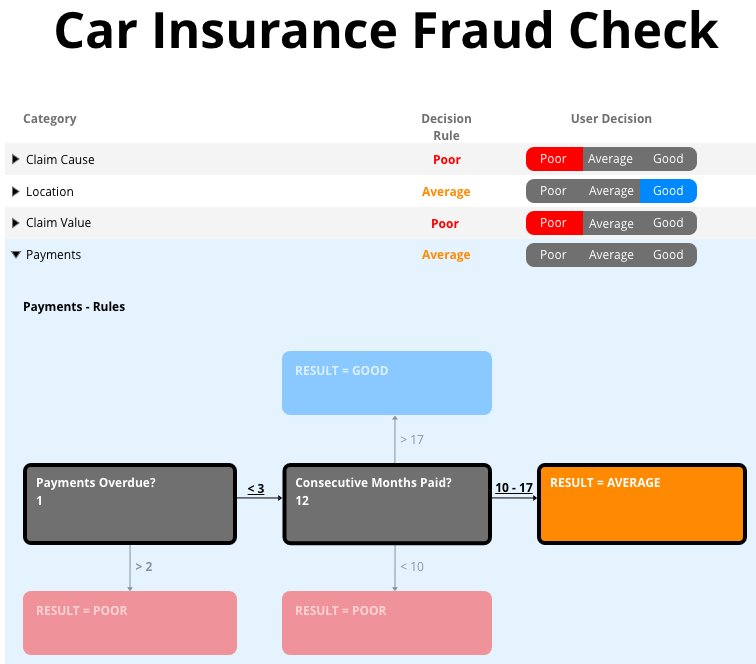

Supplement: Supplementary file 1 [file Data_Sheet_1.zip › supplementary/cropped_beslisregels_2.jpg]

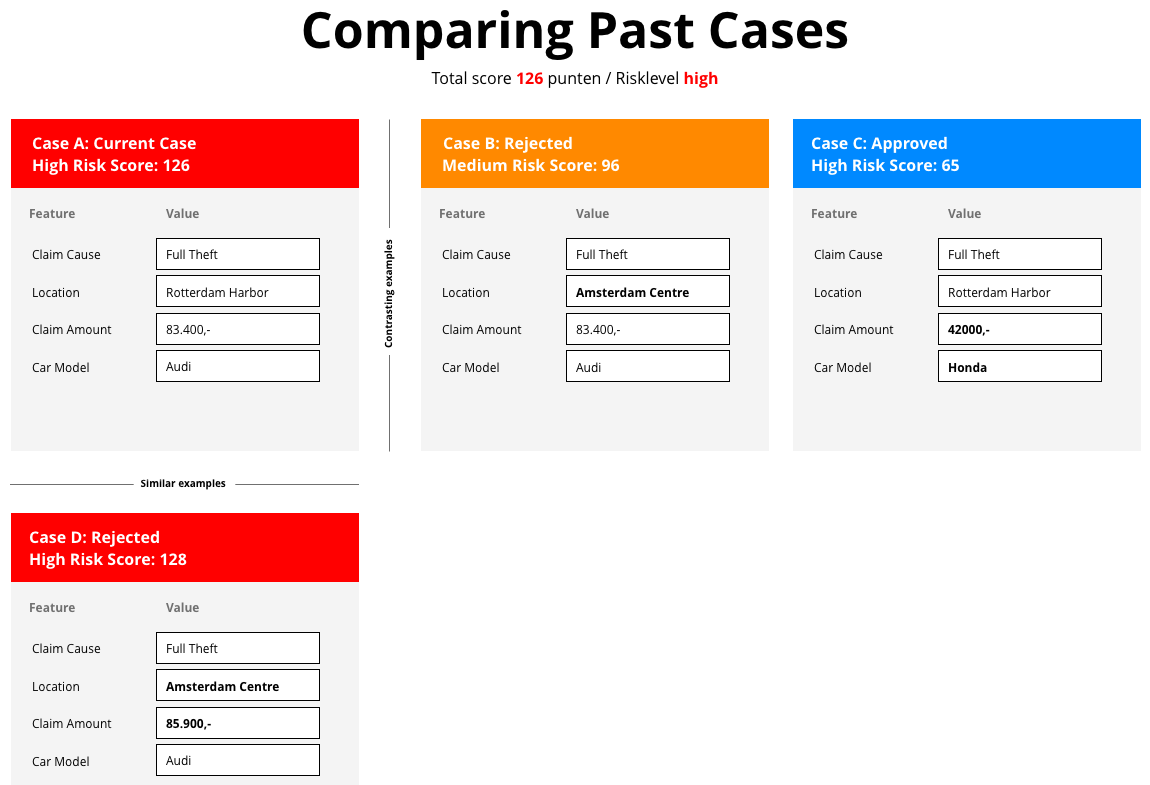

Supplement: Supplementary file 1 [file Data_Sheet_1.zip › supplementary/cropped_contrastive_0.jpg]

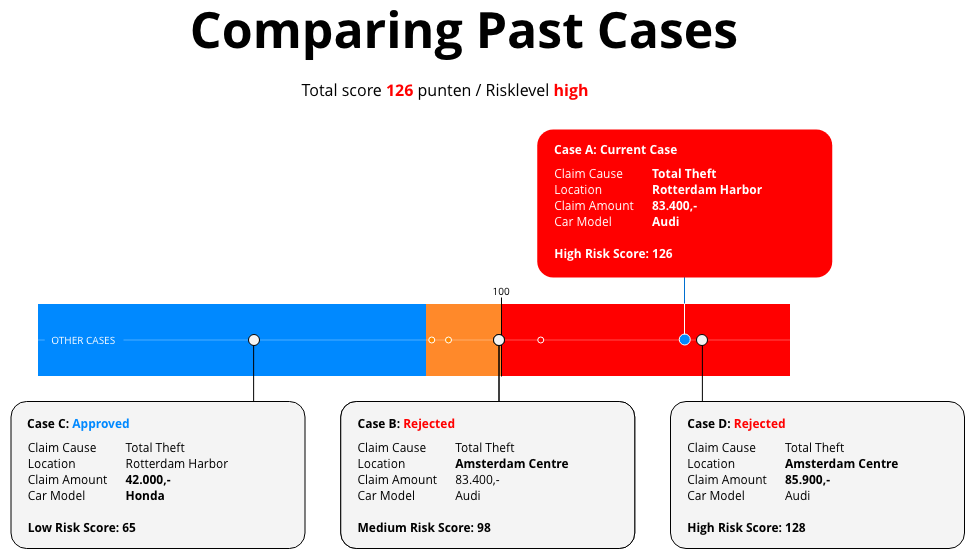

Supplement: Supplementary file 1 [file Data_Sheet_1.zip › supplementary/cropped_contrastive_2.jpg]

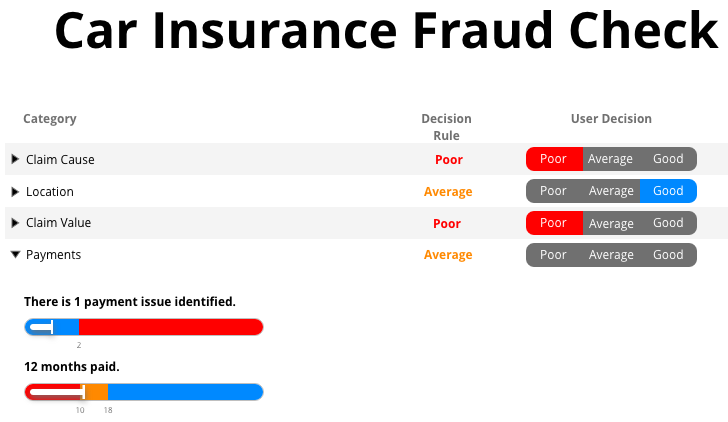

Supplement: Supplementary file 1 [file Data_Sheet_1.zip › supplementary/cropped_beslisregels_0.jpg]

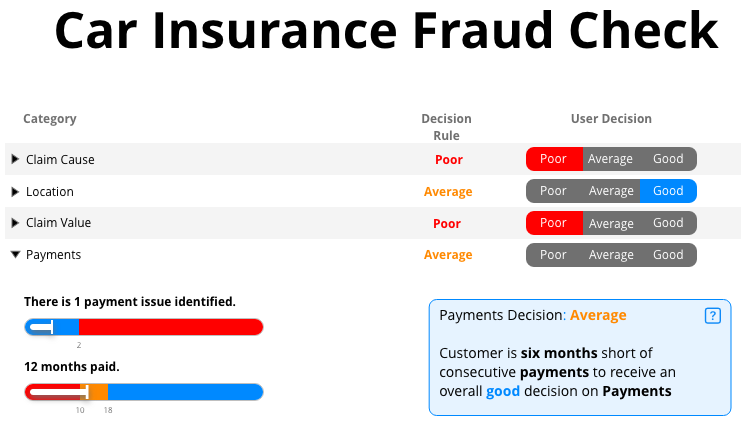

Supplement: Supplementary file 1 [file Data_Sheet_1.zip › supplementary/cropped_beslisregels_1.jpg]

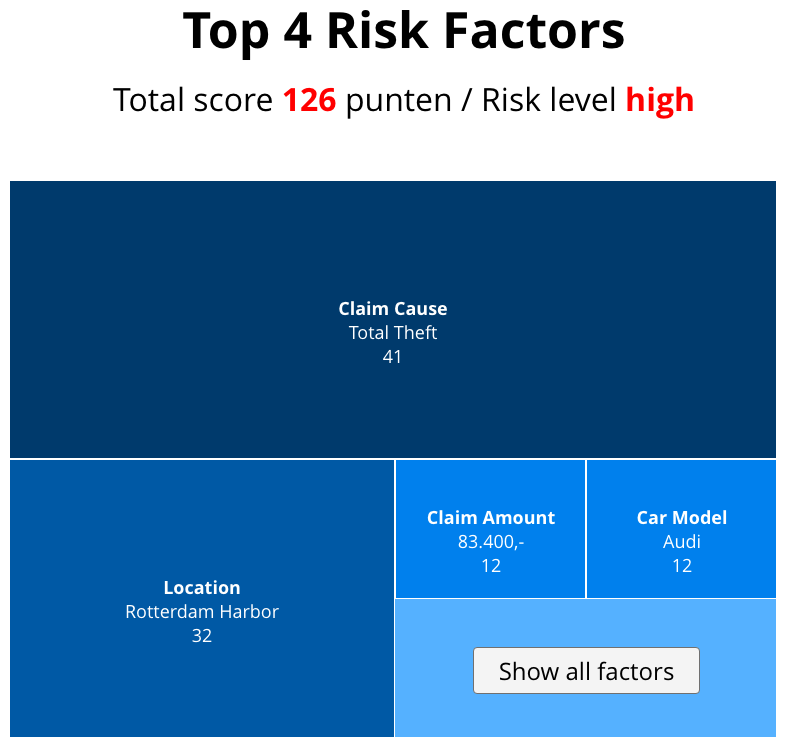

Supplement: Supplementary file 1 [file Data_Sheet_1.zip › supplementary/cropped_fi_4.jpg]

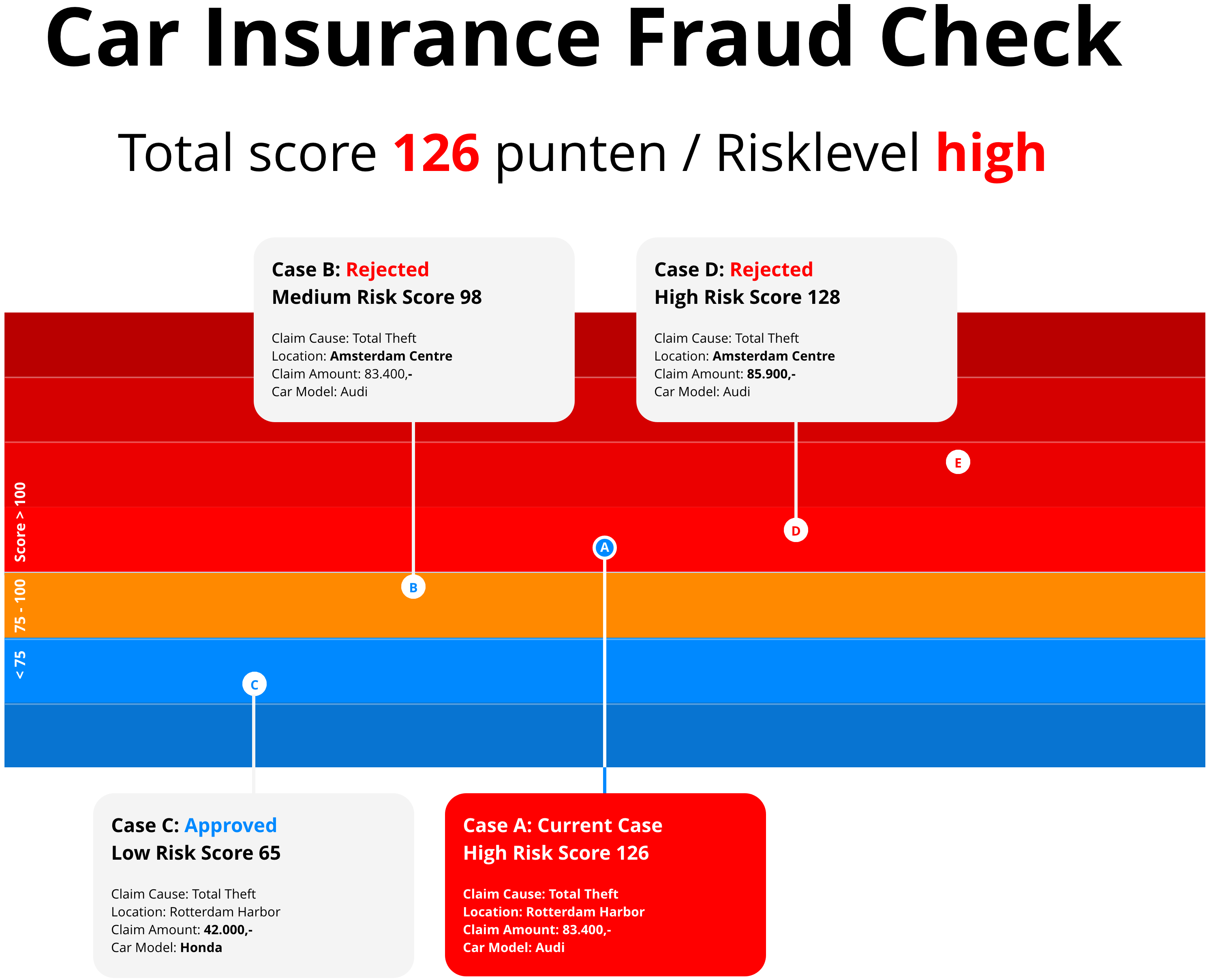

Supplement: Supplementary file 1 [file Data_Sheet_1.zip › supplementary/cropped_contrastive_3.jpg]

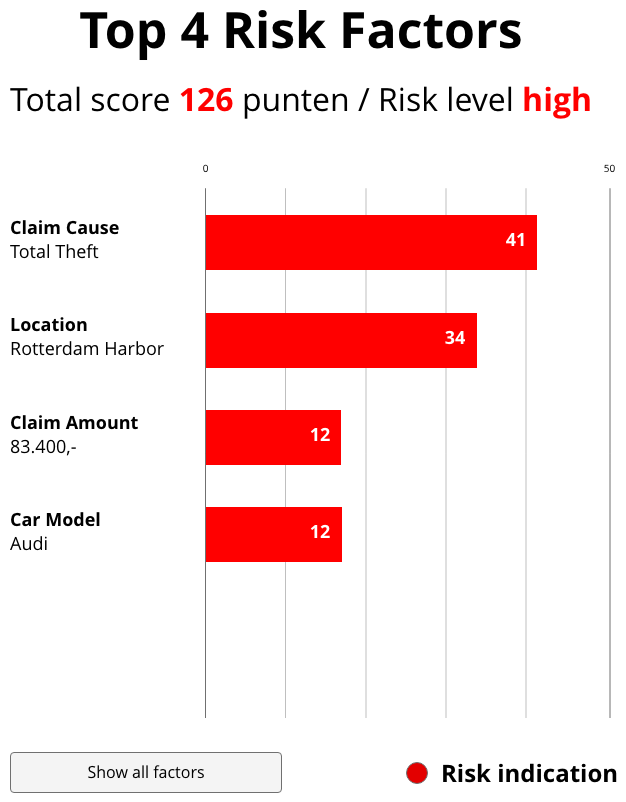

Supplement: Supplementary file 1 [file Data_Sheet_1.zip › supplementary/cropped_fi_0.jpg]

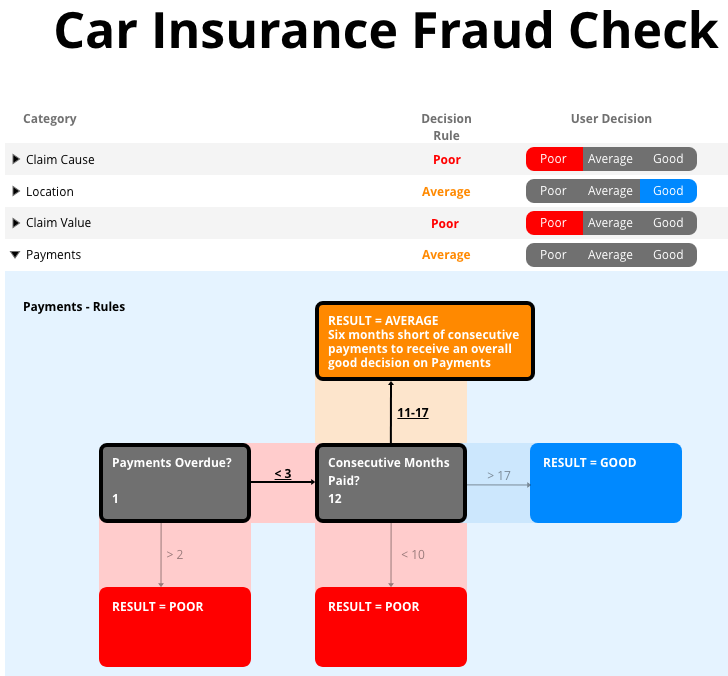

Supplement: Supplementary file 1 [file Data_Sheet_1.zip › supplementary/cropped_beslisregels_4.jpg]

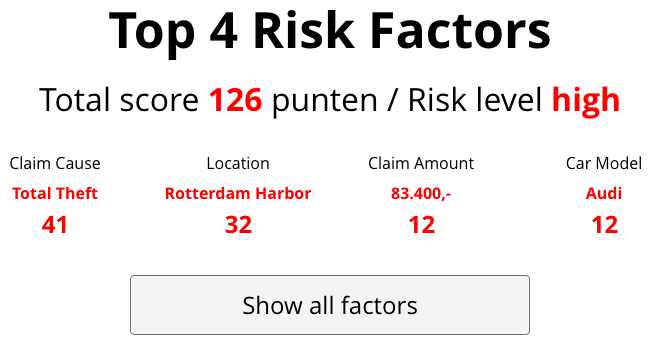

Supplement: Supplementary file 1 [file Data_Sheet_1.zip › supplementary/cropped_fi_1.jpg]

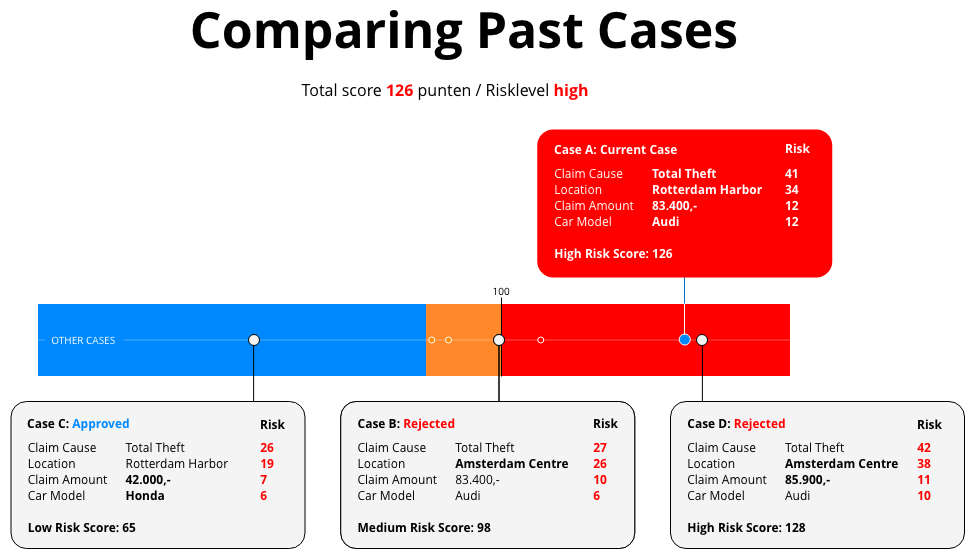

Supplement: Supplementary file 1 [file Data_Sheet_1.zip › supplementary/cropped_contrastive_4.jpg]

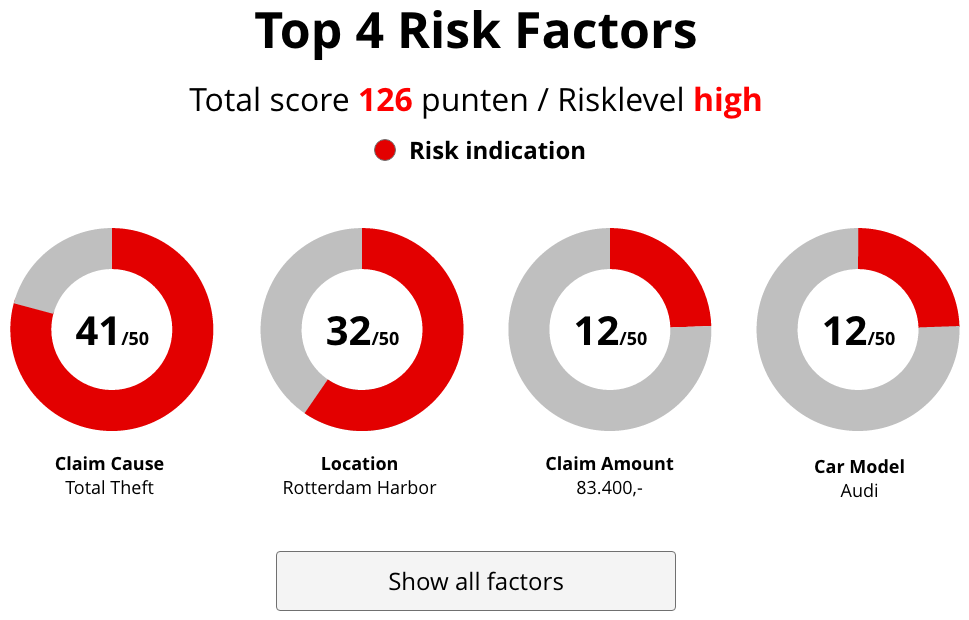

Supplement: Supplementary file 1 [file Data_Sheet_1.zip › supplementary/cropped_fi_3.jpg]

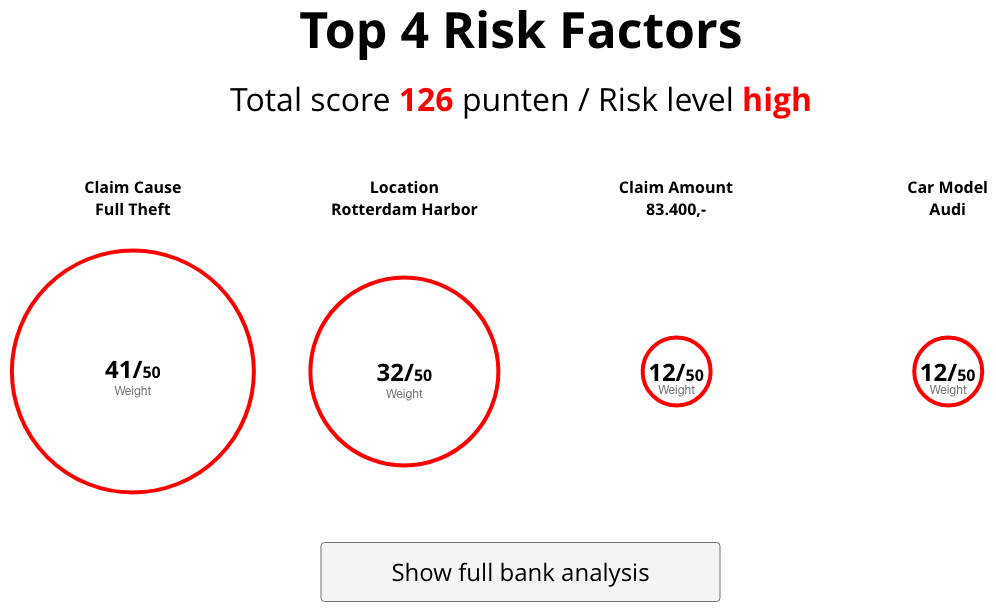

Supplement: Supplementary file 1 [file Data_Sheet_1.zip › supplementary/cropped_fi_2.jpg]
